# Metabolomic profiling of human pluripotent stem cell differentiation into lung progenitors

**DOI:** 10.1016/j.isci.2022.103797

**Published:** 2022-01-20

**Authors:** Sandra L. Leibel, Irene Tseu, Anson Zhou, Andrew Hodges, Jun Yin, Claudia Bilodeau, Olivia Goltsis, Martin Post

**Affiliations:** 1 Department of Pediatrics, University of California, San Diego, La Jolla, CA 92037, USA; 2 Translational Medicine Program, Peter Gilgan Centre for Research and Learning, Hospital for Sick Children, Toronto, Ontario M5G 0A4, Canada; 3 Sanford Burnham Prebys Medical Discovery Institute, La Jolla, CA 92037, USA

**Keywords:** Cell biology, Stem cells research, Metabolomics

## Abstract

Metabolism is vital to cellular function and tissue homeostasis during human lung development. *In utero*, embryonic pluripotent stem cells undergo endodermal differentiation toward a lung progenitor cell fate that can be mimicked *in vitro* using induced human pluripotent stem cells (hiPSCs) to study genetic mutations. To identify differences between wild-type and surfactant protein B (*SFTPB)-*deficient cell lines during endoderm specification toward lung, we used an untargeted metabolomics approach to evaluate the developmental changes in metabolites. We found that the metabolites most enriched during the differentiation from pluripotent stem cell to lung progenitor cell, regardless of cell line, were sphingomyelins and phosphatidylcholines, two important lipid classes in lung development. The *SFTPB* mutation had no metabolic impact on early endodermal lung development. The identified metabolite signatures during lung progenitor cell differentiation may be utilized as biomarkers for normal embryonic lung development.

## Highlights

• Largest metabolic changes occurred from pluripotent cells to definitive endoderm
• Differentiation to lung progenitor cell increased fatty acid metabolism
• Differentiation to lung progenitor cell decreased urea cycle/aspartate metabolism
• No significant differences between metabolites in wild type vs SPB mutant endoderm

## Graphical Abstract

**Figure:**
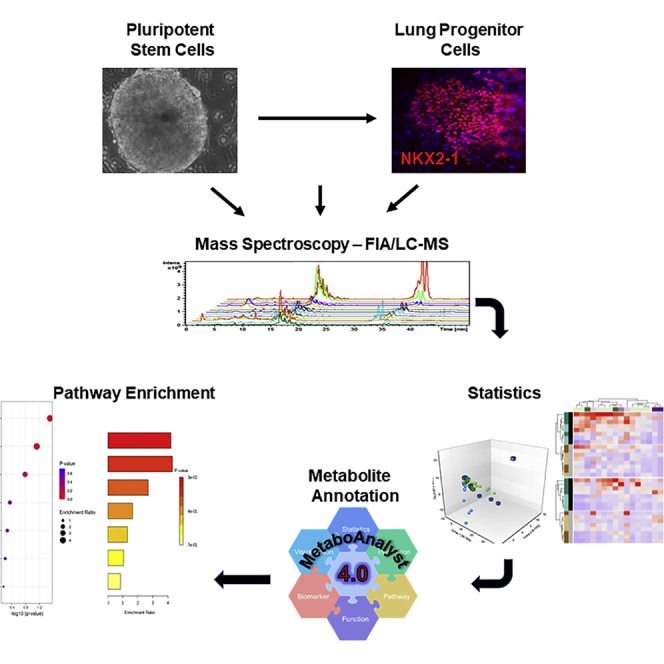

## Introduction

Respiratory distress syndrome (RDS) has a mortality rate of 13.4 per 100,000 live births and affects preterm infants with an increasing rate of morbidity as gestational age decreases (Wang et al., 2015). The etiology is the quantitative lack of functional surfactant to reduce surface tension in the background of an immature lung. Surfactant is composed of approximately 90% lipids and 10% proteins (Pérez-Gil, 2008). Surfactant proteins B and C are hydrophobic and function in the stabilization of the secreted tubular myelin to reduce surface tension in the lung. The surfactant protein B (*SFTPB*) mutation, p.Pro133GlnfsTer95 or Pro133ins2 (previously known as 121ins2), is the most common clinically relevant mutation, resulting in a frameshift, an unstable mRNA product and the absence of a protein product (Nogee, 1997). *SFTPB* deficiency affects one in a million newborn babies and is fatal without lung transplantation (Hamvas, 2006). It manifests in the postpartum period, when a newborn is unable to transition from fluid-filled to air-filled alveolar sacs due to the inability to reduce surface tension. Surfactant supplementation is to no avail and infants are kept alive on a ventilator until transplantation. Although the impact of surfactant deficiency has been successfully modeled in animals, the ability to study surfactant metabolism in a human model system and model human lung development has been challenging due to the invasive nature of primary lung tissue collection and the difficulty of maintaining human lung cell cultures *in vitro,* including alveolar epithelial cells. Immortalized human lung cell lines represent potential alternatives to primary lung tissue but have significant limitations such as low expression of surfactant proteins in the alveolar type II cells (Munis et al., 2021).

The use of human embryonic stem cells (hESC) and induced pluripotent stem cells (hiPSC) to generate human cell types has opened the field of regenerative medicine, giving investigators a reliable and reproducible *in vitro* platform to study human disease and development. Many groups have successfully used small molecules and/or growth factors to differentiate pluripotent stem cells (PSCs) into endodermal germ layer cells, mimicking embryonic lung development and achieve lung progenitors which can then be differentiated into airway and parenchymal lung cells (Dye et al., 2015; Gotoh et al., 2014; Green et al., 2011; Hawkins et al., 2017, 2020; Huang et al., 2014; Jacob et al., 2017; Konishi et al., 2016; Leibel et al., 2020; McCauley et al., 2017). These protocols have been useful in modeling human lung disease and development and could ultimately be used for future *in vivo* cell therapy (Ee and Thébaud, 2018) or screening of novel therapeutic drugs. Owing to the complexity and heterogeneity of the lung, the differentiation of PSCs into lung cells has lagged other tissue types, including the brain, heart, and liver. Lung differentiation protocols mimic the pattern of embryonic lung development and begin with definitive endoderm formation from PSCs via the Nodal signaling pathway (Zorn and Wells, 2009). After formation of the foregut tube, the endoderm is patterned into anterior and posterior directions by signals from the mesenchyme including bone morphogenetic protein (BMP), WNT, epidermal growth factor (EGF), retinoid acid (RA), and Sonic hedgehog (SHH) pathways (Kadzik and Morrisey, 2012; Rankin et al., 2016). The ventralization of anterior foregut endoderm (AFE) results in the expression of the transcription factor NKX2-1 in the primitive lung bud, marking the first step toward early lung development (Minoo et al., 2007). These NKX2-1-expressing lung progenitor cells (LPC) can spontaneously form 3D lung organoids containing proximal and distal epithelial and mesenchymal cells *in vitro* (Dye et al., 2015; Gotoh et al., 2014; Huang et al., 2014; Jacob et al., 2017; Konishi et al., 2016; Leibel et al., 2020; McCauley et al., 2017). We have previously differentiated PSCs with the *SFTPB* mutation, Pro133ins2 (previously known as 121ins2) (Nogee, 2019), into 3D human lung organoids via lung progenitor cells. We noticed reduced efficiency at the final endodermal stage (LPC) between the *SFTPB* mutant and the wild-type lines (Leibel et al., 2019). We hypothesized that this may be due to differences in the metabolome between the mutant and wild-type cell lines during the differentiation.

Metabolomics studies cellular processes in the cell, derived from chemical reactions and can be used to assess the integrity and maturation of cellular pathways (Patti et al., 2012). Biomarkers and cellular signatures have been utilized in studying lung disease such as asthma (Saude et al., 2009), acute lung injury (Stringer et al., 2016), and chronic lung disease (Nambiar et al., 2020) but have not been studied in early lung endoderm specification. Other PSC-derived tissues have undergone metabolomic investigations such as iPSC-derived cardiomyocytes (Robertson et al., 2013), neurons (Paulsen Bda et al., 2012), and hepatocytes (Jellali et al., 2020), revealing important pathways and targets for maturation, function, and therapeutics.

In the present study, we interrogated the metabolome of differentiating endodermal cells into lung progenitor cells from wild-type and *SFTPB*-deficient hiPro133 cell lines. At key stages of endoderm differentiation, including definitive endoderm (D'Amour et al., 2005), anterior foregut endoderm (Green et al., 2011) and lung progenitor endoderm (Hawkins et al., 2017), cells underwent unbiased mass spectroscopy in order to characterize six specific groups of metabolites including hexoses, amino acids, glycerophospholipids, sphingolipids, acylcarnitines, and biogenic amines. These were then analyzed to identify: 1) significant metabolic changes, differing functional pathways, and kinetics between wild-type and hiPro133 cell lines, and 2) significant biomarkers important in each step of the endodermal differentiation process from stem cell to lung progenitor.

## Results

### Directed differentiation of wild-type and hPro133 PSCs successfully derive definitive endoderm, anterior foregut endoderm, and lung progenitor cells

**Figure 1:**
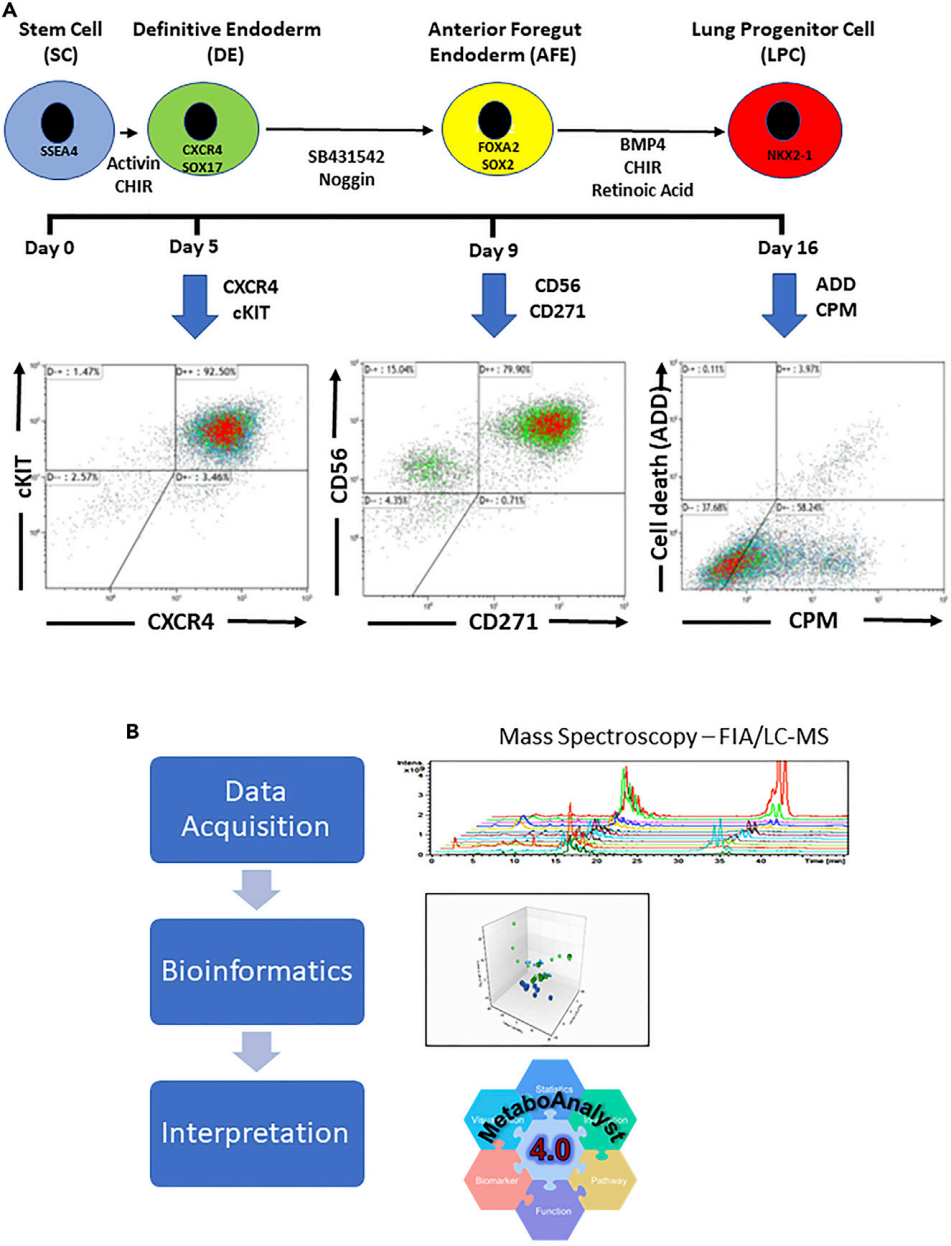
Endodermal differentiation, fluorescence-activated cell sorting, and metabolite analysis (A) Timeline of directed differentiation of human induced pluripotent stem (iPSC) and embryonic stem (ESC) cells into lung progenitor cells. Purity of cell populations at different stages of endoderm differentiation was established by flow cytometry for corresponding surface antigens (Definitive endoderm (DE): CXCR4+/cKIT+; Anterior foregut endoderm (AFE): CD56+/CD271+; Lung progenitor cells (LPC): CPM+). Cells from DE and LPC differentiations were then sorted using the same surface antigens. Each stem cell line (wild-type ESC and iPSC and two *SFTPB*-deficient (hiPro133) iPSC lines) was differentiated to LPCs four times, and each time stage-specific cell type underwent metabolome analysis (B) Workflow of metabolome analysis. Mass spectral data were acquired using flow injection analysis (FIA)-and liquid chromatography (LC)-tandem mass spectrometry. Bioinformatics was performed using three platforms, namely DIABLO, ANOVA, and subsets regression. Pathway enrichment was determined using MetaboAnalyst.

The *SFTPB*-deficient and wild-type PSC lines underwent successful endodermal differentiation (Figure 1A). Briefly, definitive endoderm (DE) was generated from PSCs by activating Nodal signaling using high-dose activin A and was characterized by the surface markers CXCR4 and cKIT (D'Amour et al., 2005; Green et al., 2011; Nostro et al., 2011). The DE was then specified into anterior foregut endoderm (AFE) through the inhibition of BMP, WNT, and TGF-B signaling using SB431542 and dorsomorphin and was characterized by the surface markers CD56 and CD271 (Brafman et al., 2013). Lung progenitor cells (LPC) were then generated by ventralizing the AFE via the reintroduction of BMP and WNT signaling as well as retinoic acid with the addition of BMP4, all-trans retinoic acid (RA), and CHIR99021. The LPCs were characterized by the expression of the transcription factor NKX2-1 and the surface marker CPM (Gotoh et al., 2014). At each stage of endodermal differentiation except AFE, the cells were sorted using antibodies against the surface markers characteristic of each stage. We used two biological replicates for the wild-type cell lines and two hiPro133 clones for each differentiation and sort. Each differentiation experiment was repeated four different times. On average, the efficiency of differentiation into CXCR4^+^/cKit^+^ DE cells was 90.7 ± 1.75% (mean ± SEM, n = 4 separate differentiations). Efficiency of AFE induction (% of CD56^+^/CD271^+^ cells) was 74.7 ± 3.4% while that of LPC (% of CPM^+^ cells) was 48.4 ± 2.7% (Figure 1A). The sorted (DE and LPC) and unsorted (AFE) cells then underwent mass spectroscopy (FIA/LC-MS/MS) and metadata bioinformatics analysis (Figure 1B).

### Metabolomic analysis of the different endodermal stages identifies uniquely altered metabolites in lung progenitor cells

To examine the metabolomic profiles associated with the different endodermal stages and cell lines, we used an untargeted metabolomics approach for hexoses (H), amino acids (AA), glycerophospholipids (GPL), sphingolipids (SPG), acylcarnitines (ACRN), and biogenic amines (BGA) (Table S1). The relative abundance of metabolites for each cell population at each stage was quantified using Sciex Analyst 1.7 software.

**Figure 2:**
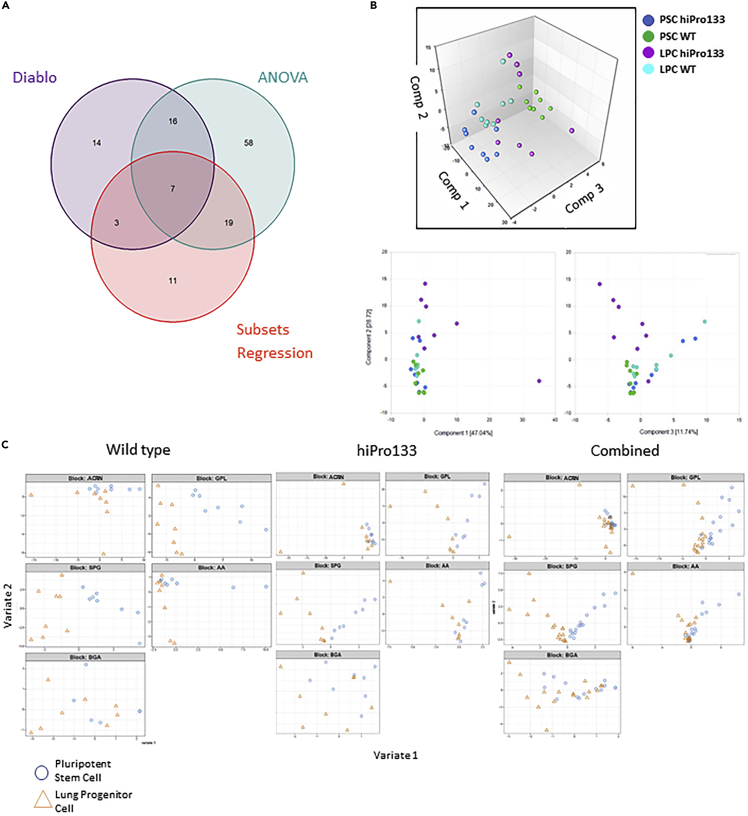
Metabolome analysis of endodermal metabolites from wild-type and hiPro133 cell lines (A) Venn diagram of significantly altered metabolites from PSC to LPC analyzed using DIABLO (PLS-DA Loadings), ANOVA (significant two-way ANOVA (hiPro133 and wild-type hPSC vs LPC)), and subsets regression (Top-10 combined signatures). Seven significantly altered metabolites from PSC to LPC were shared by all analysis platforms. (B) Principal component analysis (PCA) plot of log2 transformed data showing the variance between PSC and PSC differentiated into LPC from two cell lines (hiPro133 and wild-type) in duplicate. Bottom figures show the principal component dimension scores in 2D. Scores are calculated as a linear combination of the weights of each original metabolic group. (C) Partial least squares discriminant analysis (PLS-DA) plots of the five metabolic groups between PSC and LPC cells from wild-type, hiPro133, and combined cell lines. PSC, pluripotent stem cell; LPC, lung progenitor cell; ACRN, acylcarnitine; GPL, glycerophospholipids; SPG, sphingolipids; AA, amino acids; BGA, biogenic amines.

We performed a multivariate analysis on the metabolomic data to identify the specific signatures from the different cell lines and each differentiation stage. We used three different methods of analysis including DIABLO, ANOVA, and subsets regression. We first pooled all the data from all cell lines and examined the changes in metabolites from the beginning of the differentiation to the final product (PSC to LPC). A Venn diagram from PSC to LPC shows the number of significantly altered metabolites and which ones overlapped among the methods of analysis. Forty significantly altered metabolites were identified using the DIABLO analysis, 100 significantly altered metabolites were identified using ANOVA, and 40 significantly altered metabolites were identified using subsets regression (Figure 2A). Seven significantly altered metabolites were shared by all three analysis platforms.

To determine which metabolites had the largest effect on endodermal differentiation, we first sought to identify variations between wild-type vs hiPro133 mutant cells at various stages of endodermal differentiation (PSC to LPC). Principal component analysis (PCA) showed clustering of the wild-type and hiPro133 PSCs and wild-type and hiPro133 LPCs but clear separation of the wild-type and hiPro133 LPCs from PSCs (Figure 2B). This suggests that metabolites in pluripotent stem cells and LPCs do not differ significantly among the different cell lines. The variance was driven mainly by the differentiation stage.

We then analyzed the metabolic groups important in the variation seen in components 1 and 2 of the PCA plot between the PSC and LPC differentiation stages using DIABLO partial least squares discriminant analysis (PLS-DA). This analysis cross-compared all the samples simultaneously using the differentiation state as a categorical variable. In the PLS-DA score plots, the metabolic groups which impacted the separation of PSCs and LPCs in the wild-type cell lines were acylcarnitines (ACRN), glycerophospholipids (GPL), sphingolipids (SPG), and amino acids (AA). In the hiPro133 cell lines, separation was seen in the metabolic groups GPL, SPG, and AA. When all cell lines were combined, the only variations were in the GPL and SPG metabolic groups (Figure 2C). Thus, the metabolic groups with the most impact driving the variance between pluripotent stem cells and lung progenitor cells were glycerophospholipids and sphingolipids.

We then wanted to identify the specific metabolites in each group which had a significant impact on the variation between the differentiation stages. The top metabolites contributing to the variation at each endodermal step are found in Table 1. Loading vectors from DIABLO were analyzed where the loading weights are represented in decreasing order from bottom to top, with the bottom vectors representing the most important metabolites (Figures S1–S3). The absolute value (x axis) indicated the importance of each metabolite in defining the variations in principle component 1 from the PCA plots. In the wild-type and hiPro133-combined cell lines (Figure S1), C2 acylcarnitine was the most important ACRN for separating LPCs from PSCs, suggesting increased fatty acid beta-oxidation in LPCs. Glycerophospholipids (GPC) contributed to both PSCs and LPCs with a mix of positive and negative weights. The most important GPC contributor to LPCs was phosphatidylcholine diacyl C40:1 while the most important contributor to PSCs was phosphatidylcholine acyl-alkyl C42:5. Sphingomyelin C22:3 (SM d18:½2:3) had the greatest contribution to LPCs of all measured sphingolipids while dihydrosphingomyelin C22:2 (DHSM d18:½2:2) was important for PSCs. Of the amino acids, lysine contributed mostly to PSCs while tryptophan was important for LPCs. Finally, biogenic amines contributed little to the separation along component one, but all metabolites favored LPCs, and creatinine had the largest impact. The metabolic impact on PCA variation in wild-type cells alone and hiPro133 cells alone are found in Figures S2 and S3.

**Table 1:** Top metabolites from DIABLO analysis for every differentiation step

Step in Differentiation	MetIDQ Name	Biochemical Name
PSC to DE	PC.aa.C24.0	Phosphatidylcholine diacyl C24:0
	PC.ae.C40.1	Phosphatidylcholine acyl-alkyl C40:1
	PC.ae.C40.6	Phosphatidylcholine acyl-alkyl C40:6
	C18.1	Octadecenoylcarnitine
DE to AFE	lysoPC.a.C28.1	lysophosphatidylcholine acyl C28:1
	PC.ae.C32.2	Phosphatidylcholine acyl-alkyl C32:2
	PC.ae.C34.3	Phosphatidylcholine acyl-alkyl C34:3
	SM d18:1/18.0	Sphingomyelin C18:0
AFE TO LPC	PC.ae.C36.3	Phosphatidylcholine acyl-alkyl C36:3
	SM d18:½4.0	Sphingomyelin C24:0
	C18.1	Octadecenoylcarnitine
	Tyr	Tyrosine
PSC TO LPC	PC.ae.C32.1	Phosphatidylcholine acyl-alkyl C32:1
	PC.ae.C34.1	Phosphatidylcholine acyl-alkyl C34:1
	PC.ae.C44.6	Phosphatidylcholine acyl-alkyl C44:6
	C5.M.DC	Methylglutarylcarnitine

PSC, pluripotent stem cell; DE, Definitive endoderm; AFE, anterior foregut endoderm; LPC, lung progenitor cell.

### The largest change in metabolite expression occurs during differentiation of pluripotent cells into definitive endoderm

**Figure 3:**
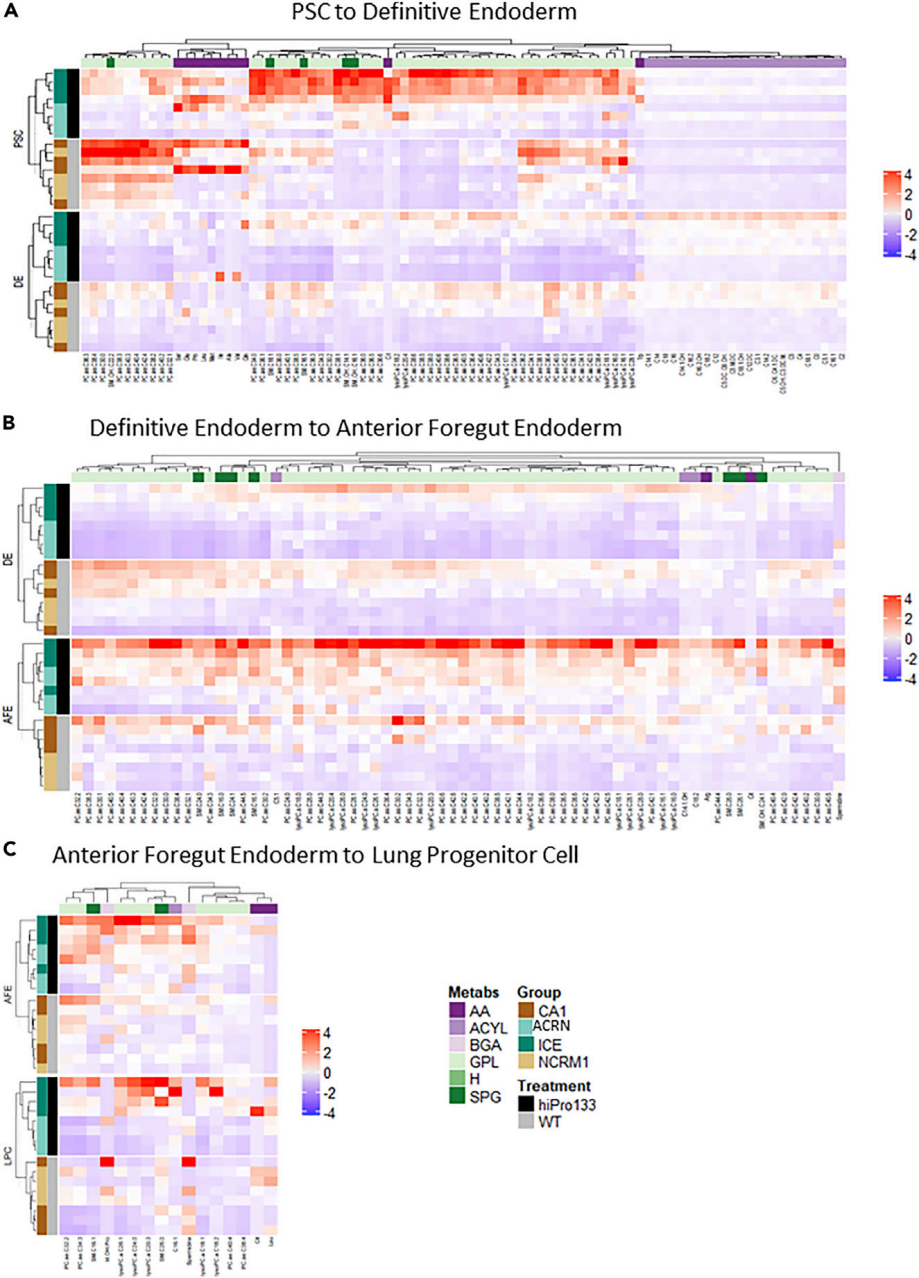
Metabolic analysis of each endodermal stage reveals differences in metabolite expression during differentiation of pluripotent stem cells into lung progenitor cells (A–C) Hierarchical clustering of filtered metabolites for hiPro133 and wild-type cell lines that significantly changed (A) from PSC to DE, (B) from DE to AFE, and (C) from AFE to LPC. N = 4 independent differentiations of two wild-type and hiPro133 cell lines. PSC, pluripotent stem cell; DE, Definitive endoderm; AFE, anterior foregut endoderm; LPC, lung progenitor cell; ACRN, acylcarnitines; GPL, glycerophospholipids; SPG, sphingolipids; AA, amino acids; BGA, biogenic amines; CA1, ESC line; NCRM1, iPSC line; ICD&ICE, hiPro133 iPSC lines. For list of metabolites, please see Table S1.

Hierarchical clustering of significant differentially expressed metabolites was calculated using ANOVA with *p* value <0.05 and showed metabolic clustering by cell groups at each differentiation step (Figure 3). Metabolites that clustered from PSC to DE were GPL and SPG along with SPG and ACRN. Expression of most metabolites was decreased from PSC to DE. Metabolites that clustered from DE to AFE were GPL and SPG, SPG and AA, and AA and ACRN, respectively. There was an increase in expression of most metabolites from DE to AFE. Metabolites that clustered from AFE to LPC were GPL and SPG. This differentiation step had the fewest amount of signature metabolites likely reflecting that the change from AFE to LPC may not result in much metabolic variance.

**Figure 4:**
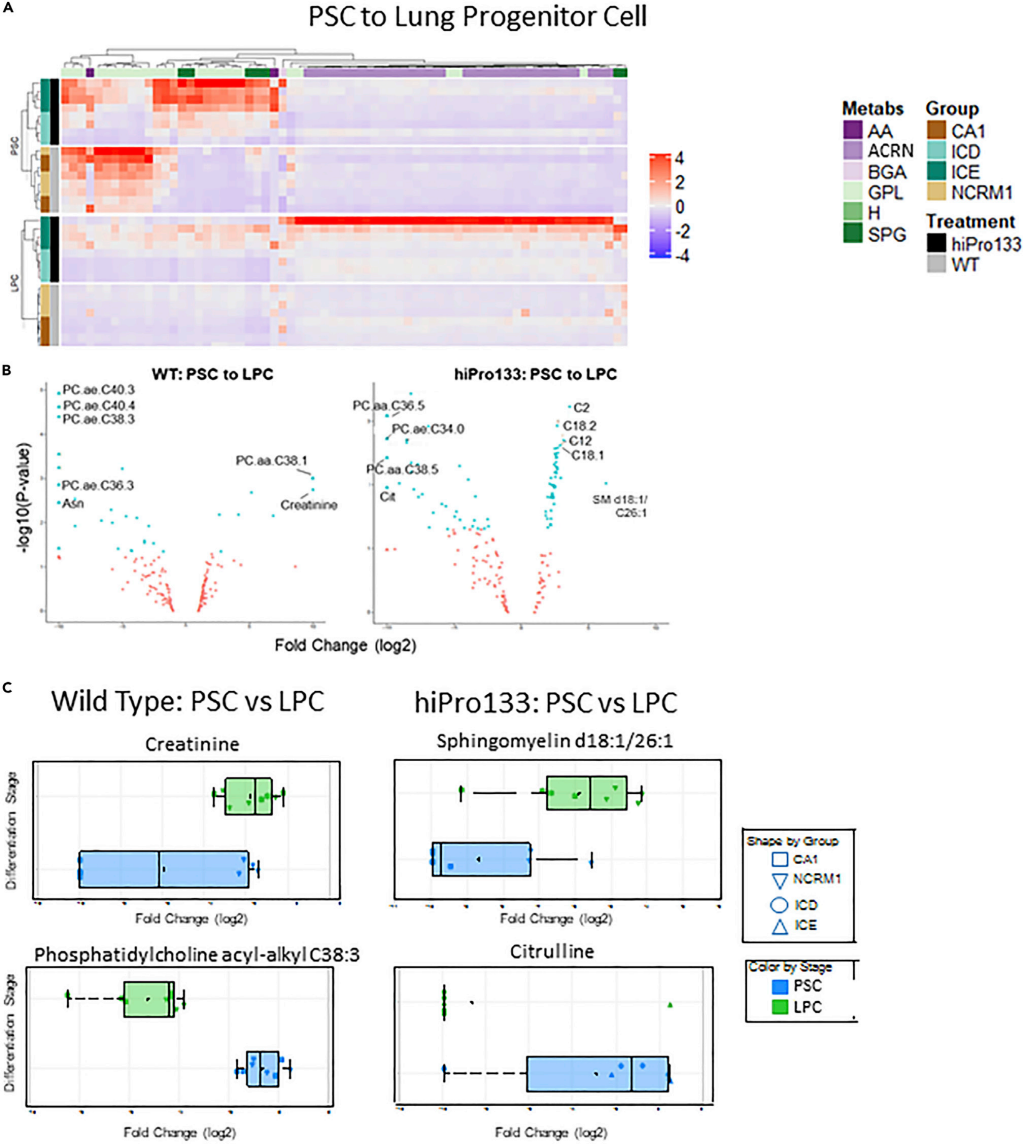
Metabolic analysis of pluripotent stem cells and lung progenitor cells identifies metabolites up- and downregulated in either wild-type or hiPro133 cells (A) Hierarchical clustering of filtered metabolites that changed significantly during differentiation from PSC into LPC. (B) Volcano plot representation of differentially expressed metabolites identified in wild-type and hiPro133 cell lines during differentiation from PSC to LPC. The fold changes are represented in a log2 scale depicted on the x axis, whereas the −log10 *p* value is depicted on the y axis (the use of −log values mean that transcripts with greater statistical significance are higher in the plot). Metabolites that are significantly up- (right side of plot) or downregulated (left side of plot) are highlighted in blue. Statistically significant cut-offs include abs (FC) ≥ 1.5 and *p* value <0.05. (C) Box-whisker plots of metabolites with the highest fold changes in the wild-type and hiPro133 lines. Distributions are shown as boxplots where the central line is the median concentration, the edges of the box are the 25th and 75th percentiles, and outliers are defined as 1.5 times the interquartile range and highlighted by + (green = LPC and blue = PSC). X axis is the log2-adjusted expression grouped per differentiation stage and cell line. Data are mean ± SD for four independent differentiations of two wild-type and two hiPro133 cell lines. PSC, pluripotent stem Cell; LPC, lung progenitor cell; ACRN, acylcarnitines; GPL, glycerophospholipids; SPG, sphingolipids; AA, amino acids; BGA, biogenic amines; H=Hexose; CA1, ESC line; NCRM1, iPSC line; ICD&ICE, hiPro133 iPSC clone lines. For list of metabolites, please see Table S1.

When differentially expressed metabolites were analyzed between PSCs and LPCs, clusters occurred in GPL and SPG (Figure 4), and most GPL and SPG metabolites were decreased in LPC while ACRN metabolites were increased in LPCs.

To evaluate which metabolites were significantly increased or decreased between PSCs and LPCs of the wild-type and hiPro133 cell lines, we used leaps regression data with a significant *p* value set at <0.05. In wild-type cells, 10 metabolites increased more than 2-fold and 29 decreased more than 2-fold when differentiated into LPCs. In the hiPro133 cells, 34 metabolites significantly increased more than 2-fold and 16 decreased greater than 2-fold. The top 10 metabolites that significantly changed more than 2-fold between PSC and LPC are shown in the volcano plots in Figure 4B and Table 2. In the wild-type cells, a mix of metabolites of the various subgroups were increased at the LPC stage while GPL metabolites represented 88% of all metabolites decreased at this stage. In the hiPro133 cells, 84% of the increased metabolites were ACRNs while 75% of the downregulated metabolites were GPLs. Box and whisker plots of the 10 most significant metabolites that changed from PSC to LPC are shown in Figures S4–S7 with representative plots of the top metabolites shown in Figure 4D. In wild-type cells, creatinine increased 17.5-fold at the LPC stage compared to undifferentiated PSC whereas phosphatidylcholine acyl-alkyl C38:3 decreased 24.5-fold. In hiPro133 cells, at the LPC stage, sphingomyelin C26:1 (SM d18:½6:1) was increased 6.3-fold when compared to undifferentiated PSCs while citrulline decreased 53.5-fold.

**Table 2:** Top 10 metabolites up- and downregulated from PSC to LPC in wild-type and hiPro133 cells

PSC VS LPC WT		PSC VS LPC hiPro133	
Down regulated	Up regulated	Down regulated	Up regulated
PC ae C38:3	Creatinine	Cit	SM C18:1/C26:1
PC ae C40:3	PC aa C38:1	Glu	C2
Asn	SM C18:1/C26:1	PC aa C36:5	C12
PC ae C36:3	SM C18:1/C26:0	PC ae C34:0	C18:1
PC ae C40:4	Kynurenine	PC aa C38:5	C18:2
PC ae C34:1	PC aa C38:6	SM C18:1/C18:1	C16:2-OH
PC ae C38:2	PC aa C36:6	PC ae C36:1	C18:1-OH
PC ae C38:4	C2	SM (OH) C18:1/C14:1	C14:2
PC ae C36:2	lysoPC a C20:4	PC aa C38:6	C4
PC ae C32:1	Trp	PC aa C38:1	C18

PC ae, Phosphatidylcholine acyl-alkyl; PC aa, Phosphatidylcholine diacyl; SM, Sphingomyelin; Asn, Asparagine; lysoPC a, lysophosphatidylcholine acyl; Trp, Tryptophan; Cit, Citrulline; Glu, Glutamate C12, Dodecanoylcarnitine; C14:2, Tetradecadienylcarnitine; C16:2-OH, Hydroxyhexadecadienylcarnitine; C18, Octadecanoylcarnitine; C18:1, Octadecenoylcarnitine; C18:2, Octadecadienylcarnitine; C18:1-OH, Hydroxyoctadecenoylcarnitine; C2, Acetylcarnitine; C4, Butyrylcarnitine.

### Metabolic differences observed between pluripotent stem cells and lung progenitors reveal pathways important in lung progenitor cell development

**Figure 5:**
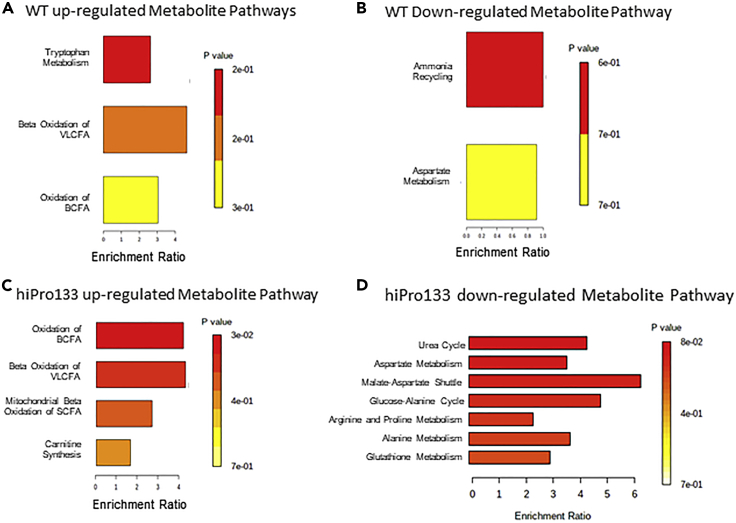
Pathway enrichment of significantly up- and down regulated metabolites between pluripotent stem cells and lung progenitors Pathway enrichment was performed using MetaboAnalyst. (A) Wild-type PSC-derived LPCs were enriched in metabolites belonging to tryptophan pathway and fatty acid oxidation. VLCFA = very long chain fatty acids. BCFA = branched chain fatty acids. (B) Metabolites that were decreased in wild-type PSC-derived LPCs belonged to ammonia and aspartate pathways. (C) hiPro133 iPSC-derived LPCs were enriched in metabolites belonging to fatty acid oxidation and carnitine synthesis. (D) Metabolites that were decreased in hiPro133 iPSC-derived LPCs belonged to urea cycle pathway. PSC, pluripotent stem Cell; LPC, lung progenitor cell

To identify similarities and dissimilarities on metabolite expression patterns among wild-type and hiPro133 LPC cells, we performed differential expression analysis and metabolite enrichment analysis with MetaboAnalyst. We identified the main biological process themes for each differentially expressed metabolite based on the number of associated metabolites and adjusted *p* values using ANOVA. For wild-type-derived LPCs, metabolites that were increased in comparison to PSCs were involved in pathways important in tryptophan metabolism and beta-oxidation of fatty acids while aspartate metabolism and ammonia recycling were suppressed. For hiPro133-derived LPCs, pathways for beta-oxidation of fatty acids and carnitine synthesis were activated, while amino acid metabolism and the urea cycle were decreased (Figure 5).

**Figure 6:**
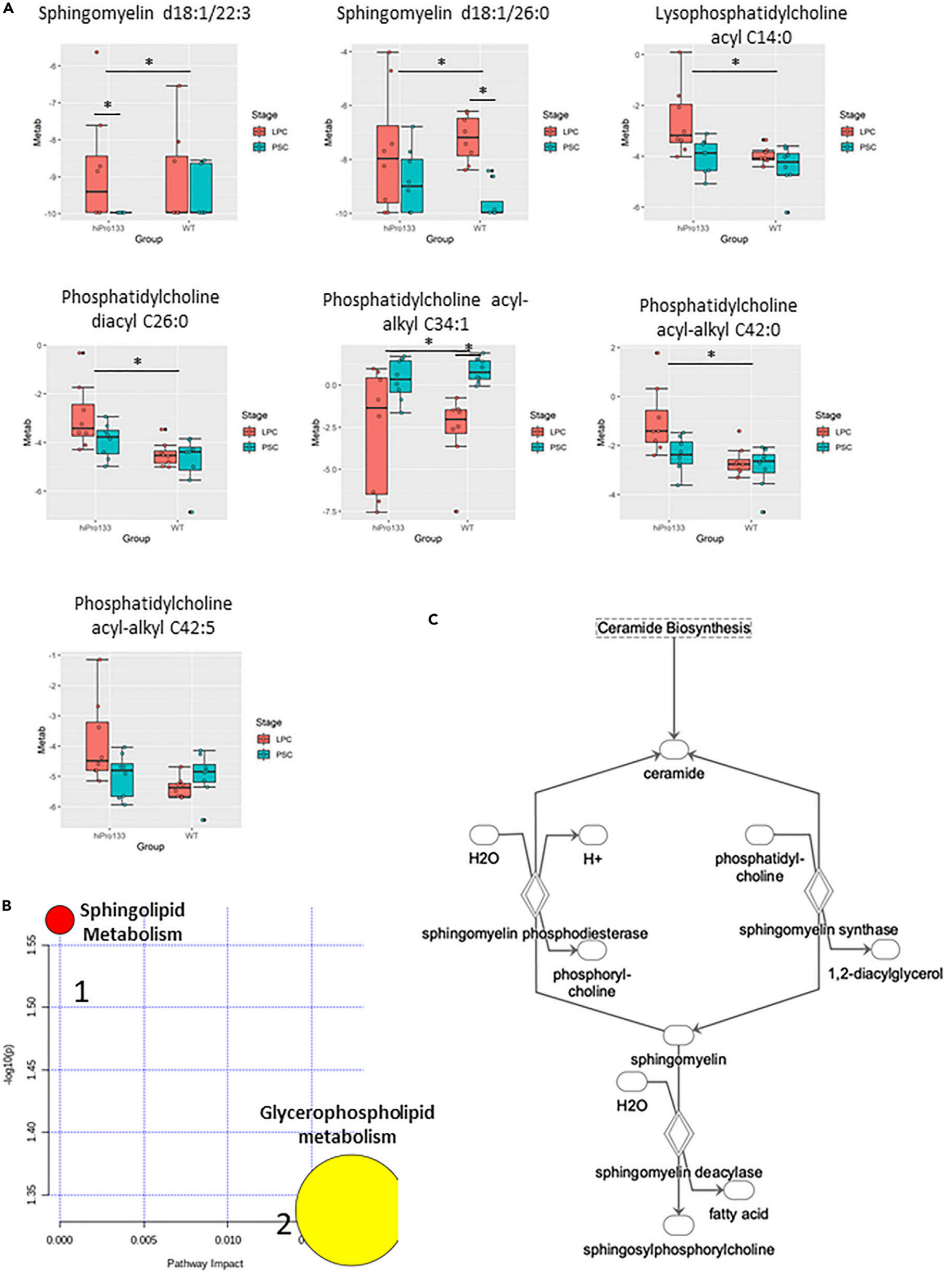
Significantly altered metabolites identified and shared by all three analysis platforms between pluripotent stem cells and lung progenitor cells (A) Seven metabolites whose peak intensity is significantly different in wild-type and hiPro133 lines from PSC to LPC (Wilcoxon rank-sum test *p* < 0.05, FDR < 0.15). Distributions are shown as boxplots where the central line is the median concentration, the edges of the box are the 25th and 75th percentiles, and outliers are defined as 1.5 times the interquartile range and highlighted by + (red = LPC and green = PSC). Y axis is the log2-adjusted expression grouped per differentiation stage and cell line. Data are mean ± SD for four independent differentiations, n = 4, ∗p < 0.05. (B) Enriched metabolic pathways of the seven top shared metabolites. The analysis was performed using MetaboAnalyst software. 1 = Sphingolipid metabolism, 2 = Glycerophospholipid metabolism. (C) Ceramide biosynthesis pathway important in PSC to LPC metabolism.

As mentioned earlier, when LPCs from all cell lines were combined, seven metabolites significantly differed from PSCs (Figure 2A). The metabolites were sphingomyelin C22:3 (SM d18:½2:3), sphingomyelin C26:0 (SM d18:½6:0), lysophosphatidylcholine C14:0, phosphatidylcholine diacyl C26:0, phosphatidylcholine acyl-alkyl C42:5, phosphatidylcholine acyl-alkyl C34:1, and phosphatidylcholine acyl-alkyl C42:0. Distributions of metabolites in boxplots using Wilcoxon rank-sum test (*p* < 0.05, FDR<0.15) showed that the sphingolipids were upregulated at the LPC stage in both wild-type and hiPro133 cell types. Lysophosphatidylcholine and phosphatidylcholine diacyl C26:0 were upregulated in only hiPro133 LPCs, and both phosphatidylcholine acyl-alkyls differed in their kinetics between the cell types, although only phosphatidylcholine acyl-alkyl C34:1 showed a significant decrease at the LPC stage in both cell lines (Figure 6A). The pathway enrichment analysis of the seven metabolites using MetaboAnalyst revealed that the sphingolipid and glycerophospholipid pathways were important at the LPC stage in both cell lines, specifically the pathways to make sphingomyelins and phosphatidylcholines (Figure 6B). These metabolites share a common pathway in ceramide biosynthesis, which has been implicated in regulating cell death (Figure 6C) (Lee et al., 2015).

### Metabolic biomarkers identified for endodermal differentiation

**Figure 7:**
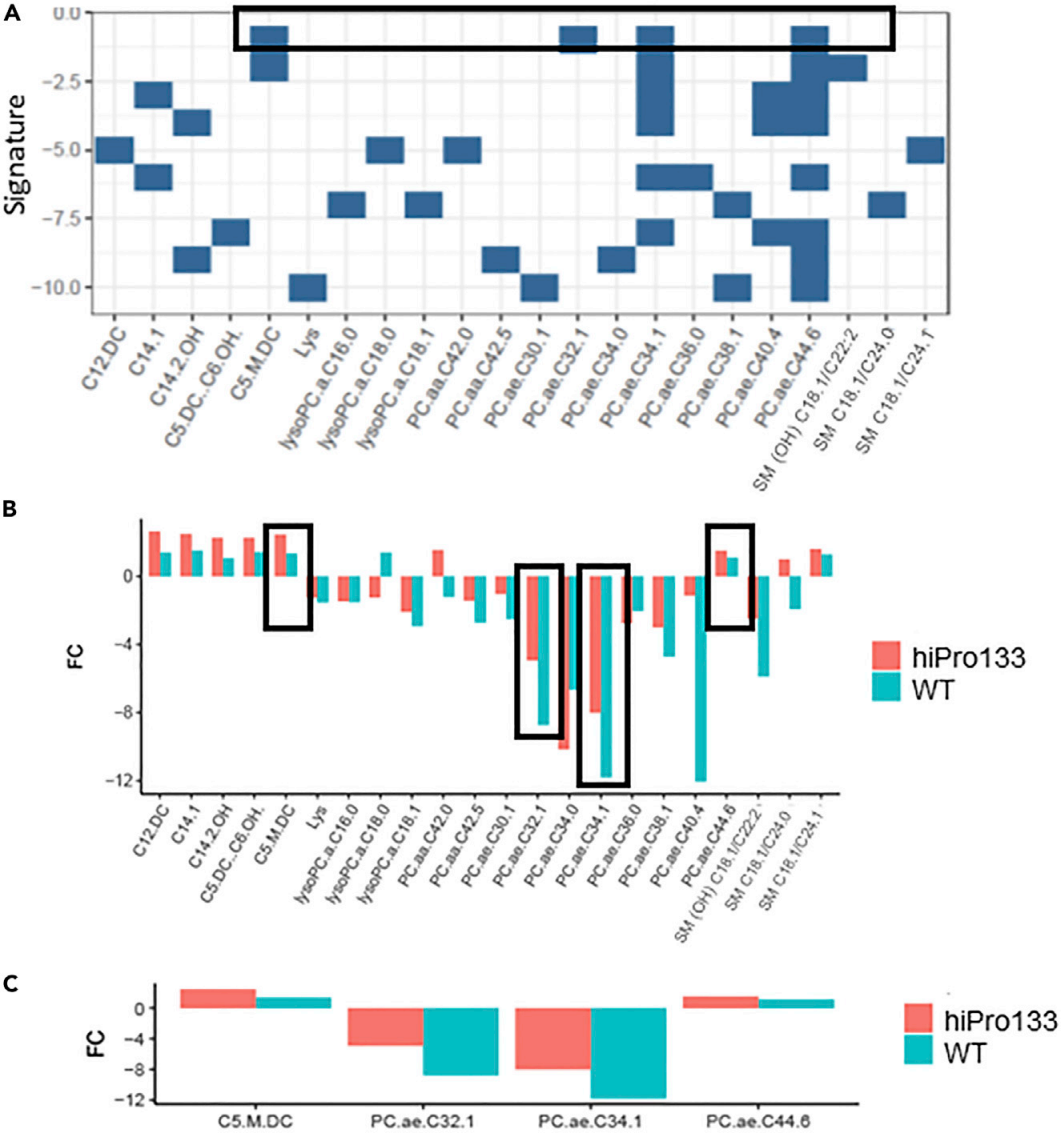
Bayesian information criterion (BIC) plots with top signatures for altered metabolites between pluripotent stem cells and lung progenitors from wild-type and hiPro133 combined and independently (A) Metabolite membership per signature (top = strongest, bottom = weakest). Black box shows top metabolites from the DIABLO analysis that contributed to the separation of PSC to LPC. Top metabolites are C5-*M*-DC (Methylglutarylcarnitine), PC.ae.C32.1 (Phosphatidylcholine acyl-alkyl C32:1), PC.ae.C34.1 (Phosphatidylcholine acyl-alkyl C34:1), and PC.ae.C44.6 (Phosphatidylcholine acyl-alkyl C44:6) in all cell lines combined. (B) Significant fold changes from PSC to LPC between wild-type and hiPro133 groups. Kinetics of selected metabolites analyzed by subsets regression. Black boxes highlight the metabolites that had the strongest metabolite memberships (see A). (C) Results from (B) in a per-signature basis as sub-figures. FC (fold change). FC > 0: metabolite upregulated and FC < 0: metabolites downregulated.

To find metabolic biomarkers that are important in the stepwise differentiation from pluripotent stem cell to lung progenitor cell, we used DIABLO to identify signature metabolites changing between two or more differentiation states in the wild-type, hiPro133, and combined cell populations. The biomarkers identified from DIABLO were compared to the subset regression using Leaps (Figure 3A). In the subset regression, each signature was given a Bayesian information criterion (BIC) score and was sorted from top to bottom (top = strongest, bottom = weakest) in the PSC to LPC analysis. The combination of the metabolites with the highest scores were the best candidates for representing biomarkers. At the LPC stage, the top metabolites were methylglutarylcarnitine (C5-*M*-DC), phosphatidylcholine acyl-alkyl C32:1 (PC.ae.C32.1), phosphatidylcholine acyl-alkyl C34:1 (PC.ae.C34.1), and phosphatidylcholine acyl-alkyl C44:6 (PC.ae.C44.6) (Figure 7A). Kinetics of the metabolites from the BIC plots showed that C5-*M*-DC and phosphatidylcholine acyl-alkyl C44:6 were increased at the LPC stage while phosphatidylcholine acyl-alkyl C32:1 and phosphatidylcholine acyl-alkyl C34:1 were decreased (Figures 7B and 7C).

We then examined the biomarkers for each step of early lung endoderm differentiation (Figure S8). We combined all the cell types and analyzed the metabolites that were highly expressed at each developmental stage regardless of genetic background. At the PSC to DE stage, the metabolites with the strongest signatures were phosphatidylcholine diacyl 24:0, phosphatidylcholine acyl-alkyl C40:1, phosphatidylcholine acyl-alkyl C40:6, and octadecenoylcarnitine. At the DE to AFE stage, they were lysophosphatidylcholine acyl C28:1, phosphatidylcholine acyl-alkyl C32:2, phosphatidylcholine acyl-alkyl C34:3, and sphingomyelin C18:0 (SM d18:1/18:0). At the AFE to LPC stage, they were phosphatidylcholine acyl-alkyl C36:3, sphingomyelin C24:0 (SM 18:½4:0), octadecenoylcarnitine, and tyrosine.

## Discussion

To our knowledge, this is the first study to use an untargeted approach to analyze the metabolome during early human endodermal differentiation, ending at the lung progenitor cell fate. Changes in cell metabolism are critical for successful lung differentiation and it was fascinating to see that the genetic background of the cell lines used (wild-type and hiPro133) did not change the metabolic signatures. The most important changes came from differentiating cells from pluripotent stem cells to lung progenitor cells. The metabolites that changed significantly included phosphatidylcholines (membrane composition and signaling), lysophosphatidylcholine (breakdown product of lipids and signaling), sphingolipids (membrane composition and signaling), and acylcarnitines (energy metabolism and fatty acid oxidation).

The greatest metabolic changes were seen at the PSC to definitive endoderm (DE) transition. The heat maps showed a sharp decrease in expression of many metabolites during DE differentiation including glycerophospholipids, sphingolipids, and amino acids, specifically serine, glutamate, proline, leucine, methionine, isoleucine, alanine, valine, and glutamine. Acylcarnitines increased during DE differentiation. This can be interpreted as a switch from glycolysis which is driven in part by amino acids to oxidative phosphorylation, driven by acylcarnitines. Pluripotent stem cells degrade glucose for ATP production via the glycolytic pathway (Shyh-Chang et al., 2013). The folate cycle is activated to maintain pluripotency via threonine-glycine catabolism (Jang et al., 2015). This results in high consumption of serine in the differentiation to DE. Recent studies examining definitive endoderm differentiation from ESCs showed that mitochondrial mass and DNA were significantly increased upon DE differentiation, along with increased ATP and reactive oxygen species. Glycolysis genes were shut off while oxidative phosphorylation genes were increased (Li et al., 2020). Open chromatin profiles of the DE stage are the most divergent from all other stages of differentiation (Kerschner et al., 2020). Our metabolomic changes are in line with these genomic and epigenomic findings. The metabolites which increased from DE to AFE were mostly glycerophospholipids. This class of lipids determine and regulate membrane composition and function and are also involved in signaling. Single-cell RNA sequencing evaluation of DE to AFE showed enrichment in the pathways for cholesterol and organic hydroxy compound metabolic processes (Kerschner et al., 2020), affirming the importance of lipid metabolism at AFE. Very little metabolites significantly changed from AFE to LPC which suggests that the stages are very similar. Early lung progenitors are probably appearing at the AFE stage and continue to differentiate into LPCs.

The metabolites that changed the most from PSC to LPC based on three different statistical interpretations were sphingolipids and glycerophospholipids, specifically sphingomyelin and phosphatidylcholine, or lecithin. Of the latter lipid class, mainly ether (alkyl-acyl) species were majorly changed. Emerging evidence suggest that ether-linked phosphatidylcholines such as plasmalogens are involved in cellular signaling and differentiation (Dean and Lodhi, 2018). Like ether lipids, sphingomyelins are major membrane constituents of lipid raft domains that are cholesterol-rich membrane regions important for signaling. Sphingomyelins are derived from ceramide and phosphatidylcholine in a reaction catalyzed by sphingomyelin synthase. However, under specific conditions, sphingomyelins are hydrolyzed to ceramide by sphingomyelinases. Ceramide is broken down by ceramidases to sphingosine that can be phosphorylated by sphingosine kinases to form sphingosine-1-phosphate (S1P). Both ceramide and S1P govern various signaling pathways related to lung cell proliferation, differentiation, survival, and senescence (Lee et al., 2015). Sphingolipids and lecithins are also critical in lung development and have long been targets for clinical application. For decades, the lecithin-sphingomyelin ratio (L/S ratio) has been used as an amniocentesis-based method for assessment of fetal lung maturity and by extension, safety of early delivery (Roux et al., 1972). However, LPCs represent the initial stage of lung epithelial development and most lecithin changes observed at this time are ether (acyl-alkyl) phosphatidylcholines and we hypothesize that the observed metabolic changes in sphingomyelins and lecithins are probably important for regulating cell proliferation, death, and differentiation that is occurring in early lung development (Whitsett et al., 2019). Sphingolipids also impact lung disease such as cystic fibrosis. Studies have found that the dysfunction of the cystic fibrosis transmembrane conductance regulator (CFTR) is largely due to aberrant sphingolipid homeostasis resulting from increased ceramide synthesis (Hamai et al., 2009; Teichgräber et al., 2008). Although we have only looked at early lung development up to the lung progenitor stage, changes in sphingolipid metabolism may be impacting lung development earlier than initially thought.

We evaluated whether *SFTPB* deficiency affects early endodermal differentiation, thus possibly predisposing affected babies to early lung cell abnormalities. The most common mutation is the p.Pro133GlnfsTer95 or Pro133ins2 mutation which results in a frameshift mutation, degradation of the abnormal mRNA product, and no protein production (Hamvas, 1997; Hamvas et al., 1995). This fatal disease affects full-term babies who present with respiratory distress syndrome, require intubation, and are ventilator-dependent until lung transplantation or death (Ballard, 1996; Hamvas et al., 1997; Nogee et al., 1993). Although this disease presents in the postnatal period, we were interested in examining whether there were differences in early development, that may impact the severity of the disease, by examining metabolite profiles between the mutant versus normal cell lines in early lung development. The metabolic pathways that were upregulated during the differentiation of PSCs to LPCs in the mutant line were fatty acid oxidation pathways and carnitine synthesis. Fatty acid oxidation is a source of energy in a developing lung, and carnitine and its esterified derivatives acylcarnitines are essential for oxidative catabolism of the fatty acids. Specific metabolites that had the highest fold changes in the mutant line were sphingomyelin and acetylcarnitine. The metabolic pathways that were downregulated during the differentiation of PSCs to LPCs in the mutant line were the urea cycle and amino acid metabolism. Although the urea cycle occurs in the liver, the metabolites involved in the urea cycle as well as nitric oxide synthesis, citrulline and glutamate, were the most down-regulated metabolites in the mutant LPCs. Glutamate is an important precursor to glutathione, an important antioxidant in the lung, therefore lower levels of this metabolite may result in increased susceptibility to oxidative stress (Rahman and MacNee, 2000). Abnormal citrulline production may lead to alveolar and vascular lung derangement due to abnormal nitric oxide levels, which along with increased surface tension and oxidate stress (Antosova et al., 2017), may exacerbate the inability of the newborn lung to function in gas exchange (Fike et al., 2014; Vadivel et al., 2010), but this needs to be evaluated at later stages in lung development. Despite these changes in metabolite expression at each endodermal stage in the hiPro133 line, there was no variation seen in the PCA or PLS-DA plots between the hiPro133 and wild-type PSCs or LPCs. Pathway enrichment of the significantly changed metabolites was similar between the wild-type and mutant lines, specifically fatty acid oxidation in the upregulated metabolite pathway overviews and urea cycle and aspartate metabolism in the downregulated metabolite pathway overviews. This implies that in early lung development, in our model system, there are no significant differences in metabolomic pathways that occur in hiPro133 vs wild-type cells. This is also confirmed in the BIC plots that examined the top signatures of metabolites at each endodermal stage for both the wild-type and hiPro133 cell lines. At the LPC stage, most of the prominent metabolites expressed the same kinetics in both cell lines, implying that these may be utilized as metabolic signatures (biomarkers) for LPC differentiation in many different cell lines. Future experiments inhibiting or over-expressing these metabolites and measuring successful LPC differentiation need to be done to confirm these findings. It may be possible to transdifferentiate PSCs directly into LPCs using a combination of these metabolites to more efficiently differentiate LPCs, skipping the DE and AFE steps.

### Limitations of the study

Limitations to this study included using a mix of ESCs and iPSCs to represent the wild-type cell populations. However, we saw them clustering in the PCA plots, suggesting little metabolic differences. There was large variability in the LPC data which may have represented more than one cell type, despite sorting the LPCs for CPM. CPM is a proxy for NKX2-1, a surface antigen that is highly expressed in the lung. However, a recent publication showed that early lung progenitors are a heterogeneous population of cells, with some requiring different levels of NKX2-1 to achieve a lung cell fate, while others didn’t express NKX2-1 at all (Kuwahara et al., 2020). Therefore, despite sorting for lung progenitor cells, we may have sorted for different subtypes of progenitors, leading to the variability in our dataset. The strengths of this study were the use of multiple analytic tools to decipher such a large dataset. It was interesting to note that different metabolites were significantly changed depending on which analysis we used, and although dozens of metabolites were found to be significantly changed, only seven were shared among all forms of statistical analysis, therefore strengthening our dataset and conclusions.

The lung progenitors generated in this study were early precursors of human lung cells. Future investigations will be required to further study the metabolomic changes in lung development and maturation, possibly in specific physiologic locations such as the airway or alveolar region or both. These may show differences in metabolite expression in surfactant protein B-deficient vs wild-type cells indicating abnormal lamellar body development in the alveolus or abnormal club cell development in the airway. Supplemental investigations such as transcriptomics and proteomics with both fetal and primary lung epithelial cultures would help to better understand the relationships between the metabolic effects observed and their functional importance.

## STAR★Methods

### Key resources table

**Table:** 

REAGENT or RESOURCE	SOURCE	IDENTIFIER
**Antibodies**		

PE conjugated mouse anti-human CXCR4	BD Biosciences	Cat# 555974, RRID:AB_396267
APC conjugated mouse anti-human cKit	Biolegend	Cat# 313206, RRID:AB_314985
PE-conjugated mouse anti-human CD56	Biolegend	Cat# 362507, RRID:AB_2563924
APC conjugated mouse anti-human CD271	Biolegend	Cat# 345107, RRID:AB_10639737
mouse anti-human CPM antibody	Wako	Cat# 014-27501, RRID:AB_2801482
Alexa Fluor 488 donkey anti-mouse IgG	Invitrogen	Cat# A-11055, RRID:AB_2534102

**Chemicals, peptides, and recombinant proteins**		

Matrigel GFR	Corning	354230
mTeSR medium	StemCell Technologies	85850
ReLeSR	StemCell Technologies	05872
Rock Inhibitor Y-27632	Tocris	1254
Accutase	Innovative Cell Technologies	AT104
RPMI1640	Gibco	118755-119
B27 supplement without vitamin A	Gibco	12587-010
1% glutamax	Gibco	35050-061
penicillin/streptomycin	Gibco	15140-122
human activin A	Stem Cell Technologies	78132
CHIR99021	Tocris	4423
IMDM:F12	Gibco	12440053
N2 supplements	Gibco	17502-048
L-ascorbic acid	Sigma	A92902
monothioglycerol	Sigma	M6145
SB431542	R&D Systems	1614
Dorsomorphin	StemCell Technologies	73634
human recombinant BMP4	R&D system	314-BP
all-trans retinoic acid (RA)	Sigma	R2625
TrypLE	Gibco	12605-028
phenyl isothiocyanate	Sigma-Aldrich	139742

**Critical commercial assays**		

AbsoluteIDQ® p180 kit	Biocrates Life Sciences AG	https://www.selectscience.net/products/absoluteidq-p180-kit/?prodid=114821

**Deposited data**		

Raw and analyzed data	This paper	http://doi.org/10.21228/M8P69K

**Experimental models: Cell lines**		

Human: NCRM1	RRID: CVCL_1 × 10^71^	NHCDR Cat# ND50028
Human: CA1	Mt Sinai Hosp-Samuel Lunenfeld Research Institute	0137
Human: Pro133ins2 clone D	CCRM	n/a
Human: Pro133ins2 clone E	CCRM	n/a

**Software and algorithms**		

Sciex Analyst 1.7	sciex	https://sciex.com
XLSTAT.2016	Addinsoft	https://www.xlstat.com
Leaps package in R	CRAN	https://cran.r-project.org/web/packages/leaps/leaps.pdf
MetaboAnalyst	Xia et al. (2009)	https://www.metaboanalyst.ca/
Data Integration Analysis for Biomarker discovery using Latent components (DIABLO)	Singh et al. (2019)	http://mixomics.org/mixdiablo/
prism 6.0	GraphPad Software	https://www.graphpad.com/scientific-software/prism/

**Other**		

Beckman Coulter Gallios flow cytometer	Beckman Coulter	https://www.beckman.com/resources/videos/products/gallios-overview
Sciex Q-Trap 5500 mass spectrometer	Sciex	https://sciex.com/products/mass-spectrometers/triple-quad-systems/triple-quad-5500-system

### Resource availability

#### Lead contact

Further information and requests should be directed to and will be fulfilled by the lead contact, Sandra L. Leibel (saleibel@health.ucsd.edu).

#### Materials availability

This study did not generate new unique materials.

### Experimental model and subject details

#### Human embryonic stem cell and human induced pluripotent stem cell lines

Usage of human pluripotent cell lines was in accordance with guidelines provided by the National Institutes of Health (NIH) and the Stem Cell Oversight Committee of The Canadian Institute of Health Research (CIHR). The human embryonic stem cell line CA1 (male), the sendai generated human dermal fibroblast wild-type iPSC line hiNCRM1 (male) and the sendai generated human dermal fibroblasts hiPro133 iPSC line (Leibel et al., 2019) (clones D and E - female) containing the *SFTPB* mutation Pro133ins2, were cultured on matrigel coated (Matrigel GFR; Corning, #354230) plates in mTeSR medium (StemCell Technologies #85850). Medium was changed daily, and cells were passaged using ReLeSR (StemCell Technologies #05872) every 5–7 days. Cultures were maintained in an undifferentiated state in a 5% CO_2_/ambient air environment.

Authentication of Cell Lines is based on guidelines from the International Cell Line Authentication Committee (iclac.org): Authentication testing was performed on established cell lines regardless of the application. The pluripotency of the ESC and iPSC cell lines were confirmed through immunofluorescence for pluripotent markers (Oct4, Nanog, TRA1-81, SOX2, TRA 1-60) and normal karyotype was also confirmed prior to use. The CA1 and hiPro133 SFTPB mutant lines were previously published (Leibel et al., 2019).These cell lines have been validated by immunofluorescence for markers of pluripotency, karyotype and genomic analysis if applicable. Early passages at the time of verification of pluripotency and karyotype are cryopreserved and those stocks utilized for further experimentation.

Different cell lines and derivatives were never manipulated together to avoid the possibility of cross-contamination. Furthermore, all cell lines were used at early passage. Upon reaching higher passage number, cells were discarded, and cultures restarted from early passage cryovials. Monthly screening for mycoplasma using the MyoSEQ ThermoFisher Mycoplasma Detection kit was performed, and new cells/cell lines were maintained in quarantine until confirmed to be mycoplasma negative.

### Method details

#### Directed differentiation of hESC and hiPS cells to lung progenitor cells

All human iPSC differentiations were carried out in a 5% CO_2_/5% O_2_ environment. When the human PSCs reached 70% confluence (Day 0), the cells were incubated in 10 μM of the Rock Inhibitor Y-27632 (Tocris, #1254) for one hour and then exposed to accutase (Innovative Cell Technologies # AT104) for 20 minutes at 37°C (Gotoh et al., 2014). The detached PSCs were then dissociated into single cells via gentle pipetting, and then seeded onto thick matrigel-coated plates [1:1 mixture of Matrigel GFR and RPMI1640 (Gibco, #118755-119)] at a density of 1.75 × 10^5^ cells/cm^2^ in definitive endoderm (DE) basal medium made up of RPMI1640, 1× B27 supplement without vitamin A ((Gibco, #12587-010), 1% HEPES, 1% glutamax (Gibco, #35050-061) and 50 U/mL of penicillin/streptomycin (Gibco, #15140-122). This basal medium was supplemented with 100 ng/mL of human activin A (Stem Cell Technologies, #78132), 1 μM of CHIR99021 (Tocris, #4423), and 10 μM of Y-27632. On days 2–4, only activin was supplemented. On Day 4, the medium was changed to anterior foregut endoderm (AFE) induction medium. AFE basal medium consisted of 3:1 IMDM:F12 (Gibco #12440053), 1× B27 and 1× N2 supplements (Gibco, #17502-048), 50 U/mL of penicillin/streptomycin, 0.25% BSA, 0.05 mg/mL of L-ascorbic acid (Sigma, #A92902), and 0.4 mM of monothioglycerol (Sigma, #M6145) (Green et al., 2011). This medium was supplemented with 10 μM SB431542 (R&D Systems) and 2 μM Dorsomorphin (StemCell Technologies, #73634) for 3 days. On Day 7, the medium was changed to lung progenitor cell (LPC) induction medium, containing the AFE basal medium supplemented with 10 ng/mL of human recombinant BMP4 (R&D system, #314-BP), 0.1 μM of all-trans retinoic acid (RA) (Sigma, #R2625) and 3 μM of CHIR99021. Media was changed every 2 days for 9–11 days.

#### FACS sorting and analysis of definitive endoderm, anterior foregut endoderm and lung progenitor cells

At each differentiation step, DE, AFE and LPC, cells were analyzed by FACS for stage specific markers. The differentiated cells were harvested with TrypLE (Gibco #12605-028) at 37°C for 5-10 minutes and collected by centrifugation at 300 g for 5 minutes at room temperature. The cell pellets were resuspended in 3% (v/v) FBS in PBS, cell aggregates were removed using a cell strainer with a 40-μm pore size and single cells were collected. For surface antigens, the cells were incubated with primary antibodies for 30 minutes on ice with gentle shaking, washed twice with 3% (v/v) FBS in PBS, and if necessary, incubated with the secondary antibodies for 30 minutes followed by two more rinses with 3% (v/v) FBS in PBS. For DE cells, we used PE conjugated mouse anti-human CXCR4 (BD Biosciences #555974) and APC conjugated mouse anti-human cKit (Biolegend # 313206) antibodies; AFE cells were stained with PE-conjugated mouse anti-human CD56 (Biolegend # 362507) and APC conjugated mouse anti-human CD271 (Biolegend # 345107) antibodies (Brafman et al., 2013) and LPCs we identified using mouse anti-human CPM antibody (Gotoh et al., 2014) (Wako, # 014-27501). Alexa Fluor 488 donkey anti-mouse IgG (Invitrogen # A11055) was used as the secondary antibody against primary CPM antibody. The cells were either sorted using the BD aria FACS sorter at the Sick Kids FACS core and/or analyzed using a Beckman Coulter Gallios flow cytometer. Unstained controls were used to set up gates for FSC and SSC and double stained cells underwent fluorescence minus one (FMO) to ensure accurate gating. Sorted cells were collected and the spun down into a cell pellet and stored in −80°C degrees for further processing. The endodermal differentiations and cell sorts were repeated at four different times with two biological repeats.

#### Metabolomic mass spectrometry

Samples were analyzed for 180 metabolites using the AbsoluteIDQ® p180 kit from Biocrates Life Sciences AG (Innsbruck, Austria) at the Analytical Facility for Bioactive Molecules (The Hospital for Sick Children, Toronto, Canada). Collected cell pellets were resuspended in 15 % ice-cold 10 mM Phosphate Buffer (PB) + 85 % EtOH as per AbsoluteIDQ® p180 kit protocol. Resuspended cells were subjected to three rounds of sonication and rapid freeze thawing. Final suspension was then centrifuged at 20,000 × g for 10 minutes at 4°C. A small aliquot was taken for protein assay, and the remainder of the supernatant was used for metabolic analysis. Samples, standards, and controls (10 mL each) were added to the Biocrates 96-well filter plate with internal standard, dried under nitrogen, derivatized with phenyl isothiocyanate (Sigma-Aldrich # 139742), and then extracted with methanol, as per kit instructions. Extracted samples were analyzed by two separate tandem mass spectrometry (MS/MS) methods. Flow injection analysis (FIA)-MS/MS was used for analyzing acylcarnitines, glycerophospholipids and sphingolipids. Liquid chromatography (LC)-MS/MS using a reversed phase C18 column was used to probe for amino acids and biogenic amines. Both analytic measurements were performed using a Sciex Q-Trap 5500 mass spectrometer. Data were acquired and analyzed using Sciex Analyst 1.7 software.

#### Statistical analysis

The metabolomic multivariate data analysis was performed using XLSTAT.2016 software (Addinsoft) and MetaboAnalyst (Chong et al., 2018). Unsupervised principal component analysis (PCA) and supervised partial least squares-discriminant analysis (PLS-DA) were run to uncover significant variations between the endodermal differentiation steps and cell lines (wild-type and hiPro133) using Data Integration Analysis for Biomarker discovery using Latent components (DIABLO) (Singh et al., 2019). Each sample was assigned a score on each principal component (PC) dimension. The weights of each of the original variables were stored in loading vectors associated to each PC. The first PC (component #1) was defined as the linear combination of the original variables that explained the greatest amount of variation. The second PC (component #2) was then defined as the linear combination of the original variables that accounted for the greatest amount of the remaining variation to the first component. Component #3 was defined likewise. Discriminating metabolites were further identified using loading weights indicating the variable importance (VIP) for each metabolite in the PLS-DA projection space. Subset regression was performed using Leaps package in R (https://cran.r-project.org/web/packages/leaps/leaps.pdf) to predict combinations of metabolites best predicting separated endodermal and cell line groups. Student's t-test was performed by prism 6.0 (GraphPad Software, San Diego) using p-values of less than 0.05 for identifying statistical significance. Metabolites with significant changes in the groups (P value <0.05 and VIP >1) were selected as discriminating metabolites. Finally, pathway analyses of these potential discriminating metabolites was performed using MetaboAnalyst (Xia et al., 2009) which identified significantly enriched pathways based on the sets of metabolites. Where appropriate, we employed log base 2 normalization on the metabolite data to reduce outlier effects on model behaviors.

## Data Availability

Metabolomic data has been deposited at metabolomics work bench and are publicly available as of the date of publication. Accession numbers are listed in the key resources table. This paper does not report original code. All code utilized in the paper is available online and listed in the key resources table. Any additional information required to reanalyze the data reported in this paper is available from the lead contact upon request.
