# Supplementary material for: Metabolomic profiling of human pluripotent stem cell differentiation into lung progenitors
Source: iScience. 2022 Jan 20;25(2):103797. doi: 10.1016/j.isci.2022.103797 (PMC8850758; doi:10.1016/j.isci.2022.103797)
Supplement: Document S1. Figures S1–S8 and Table S1 [file mmc1.pdf]

iScience, Volume *25*

Supplemental information

Metabolomic profiling of human pluripotent stem cell differentiation into lung progenitors

Sandra L. Leibel, Irene Tseu, Anson Zhou, Andrew Hodges, Jun Yin, Claudia Bilodeau, Olivia Goltsis, and Martin Post

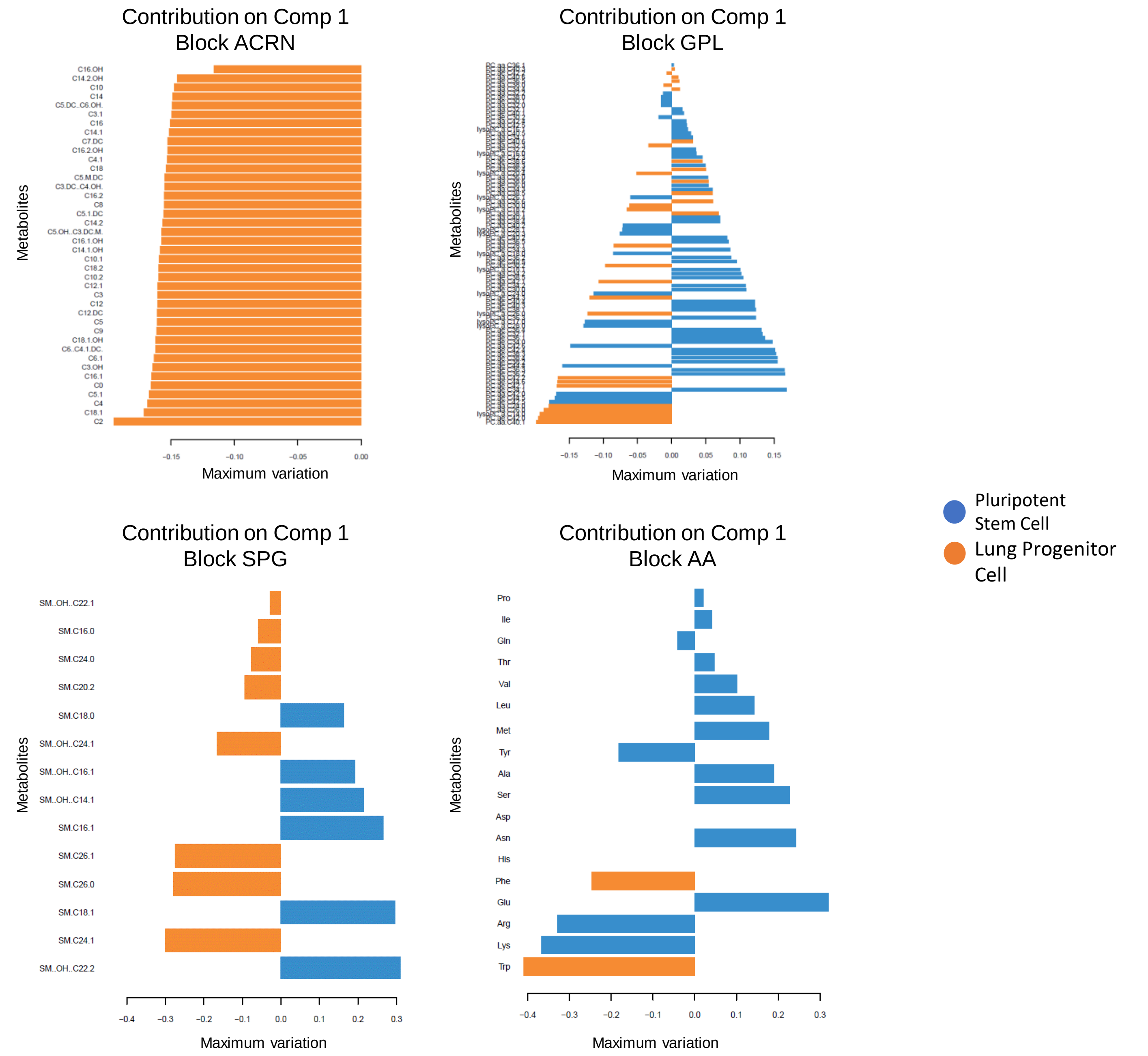

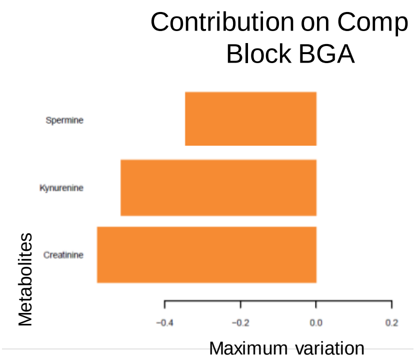

Figure S1: Loading vectors from DIABLO showing the variant metabolites from PSC to LPC from component 1 of the PCA plot for all cell lines combined. The bar length represents the linear discriminant analysis (LDA) score as a measure of significance. In the ACRN and BGA metabolite groups, significantly changed metabolites are all found in the LPC differentiation state. C2 acylcarnitine and creatinine were the most significant. In the GPL and AA metabolite groups, the metabolites were significantly altered in PSC and LPC cells in both directions with tryptophan and phosphatidylcholine acyl-alkyl C40:1 being the most significantly changed at LPC. In the SPG group, metabolites expressed significantly in PSCs are weighted towards the right direction while those expressed significantly in LPC are weighted towards the left. Dihydroxysphingomyelin C22:2 (DHSM d18:1/22:2) was the most significant. ACRN =acylcarnitine; GPL=glycerophospholipids; SPG=sphingolipids; AA=amino acids; BGA=biogenic amines

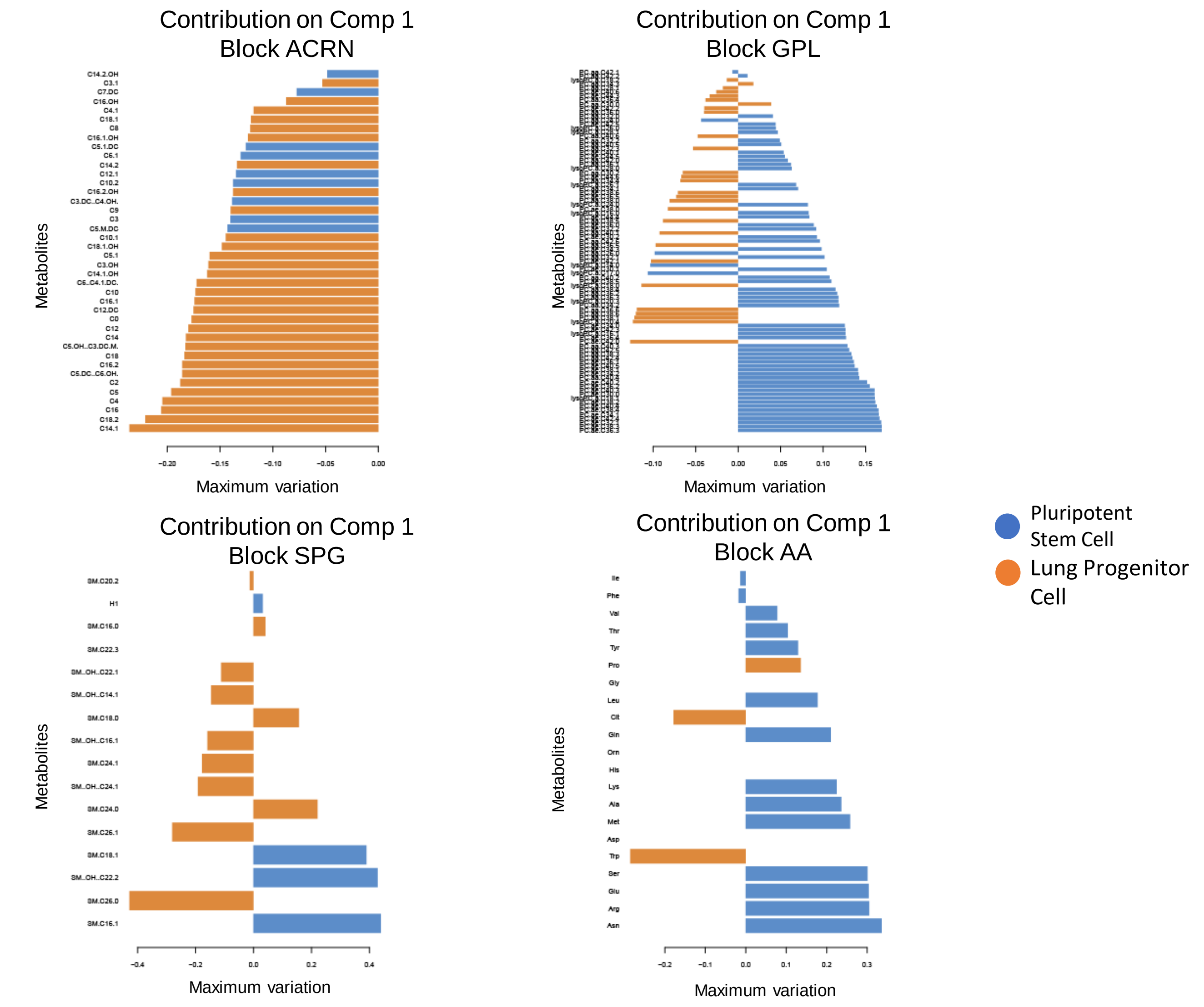

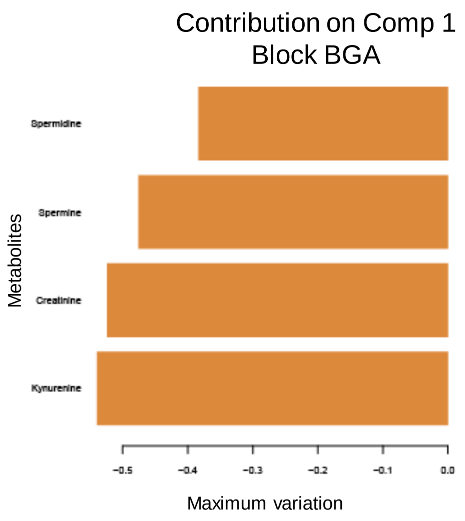

Figure S2: Loading vectors from DIABLO showing the variant metabolites from PSC to LPC from component 1 of the PCA plot for wild-type cell lines. In the ACRN, GPL, SPG and AA metabolite groups, metabolites were significantly changed in both PSCs and LPCs. The greatest weights (bottom bars of each block) were in the PSC cells in all metabolic blocks except for ACRN in which most significant changes were in the LPCs. In the BGA group, the significant metabolite changes were all found in LPCs. ACRN=acylcarnitine; GPL=glycerophospholipids; SPG=sphingolipids; AA=amino acids; BGA=biogenic amines.

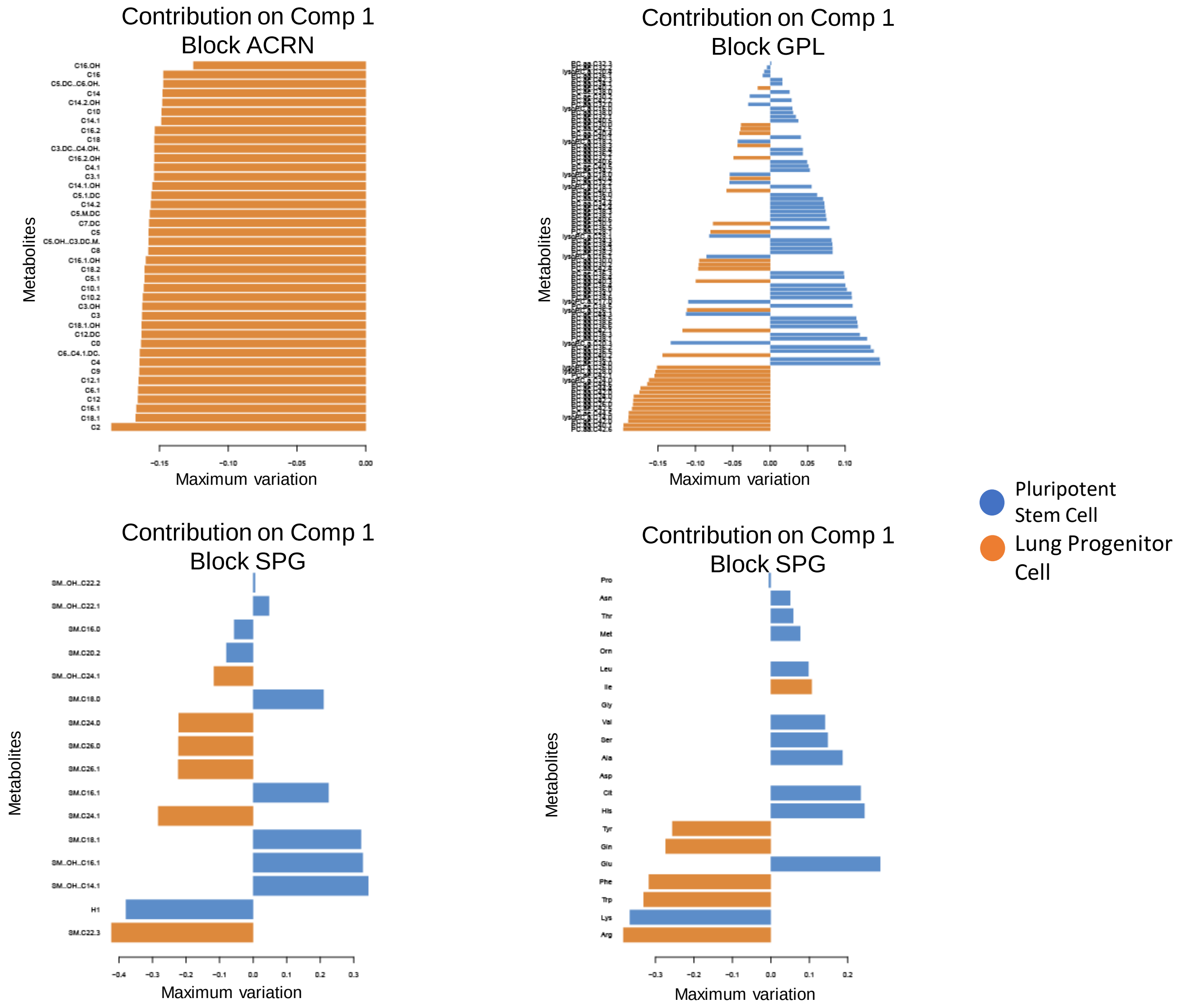

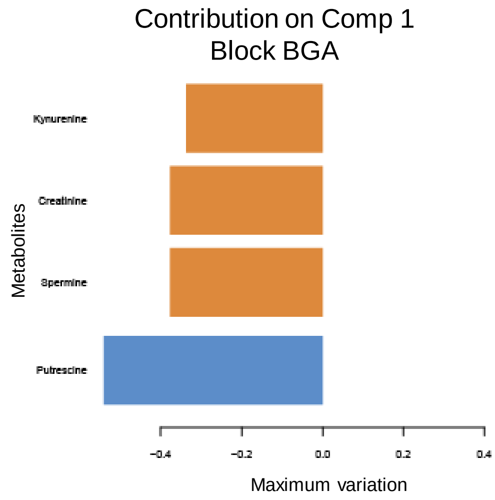

Figure S3: Loading vectors from DIABLO showing the variant metabolites from PSC to LPC from component 1 of the PCA plot for hiPro133 cell lines. In the ACRN metabolite group, significantly changed metabolites are all found in LPCs with C2 acyl carnitine being the most significant. In the GPL, SPG, AA and BGA groups, metabolites were significantly changed in both PSCs and LPCs. ACRN=acylcarnitine; GPL=glycerophospholipids; SPG=sphingolipids; AA=amino acids; BGA=biogenic amines.

S4 WT Up-regulated metabolites

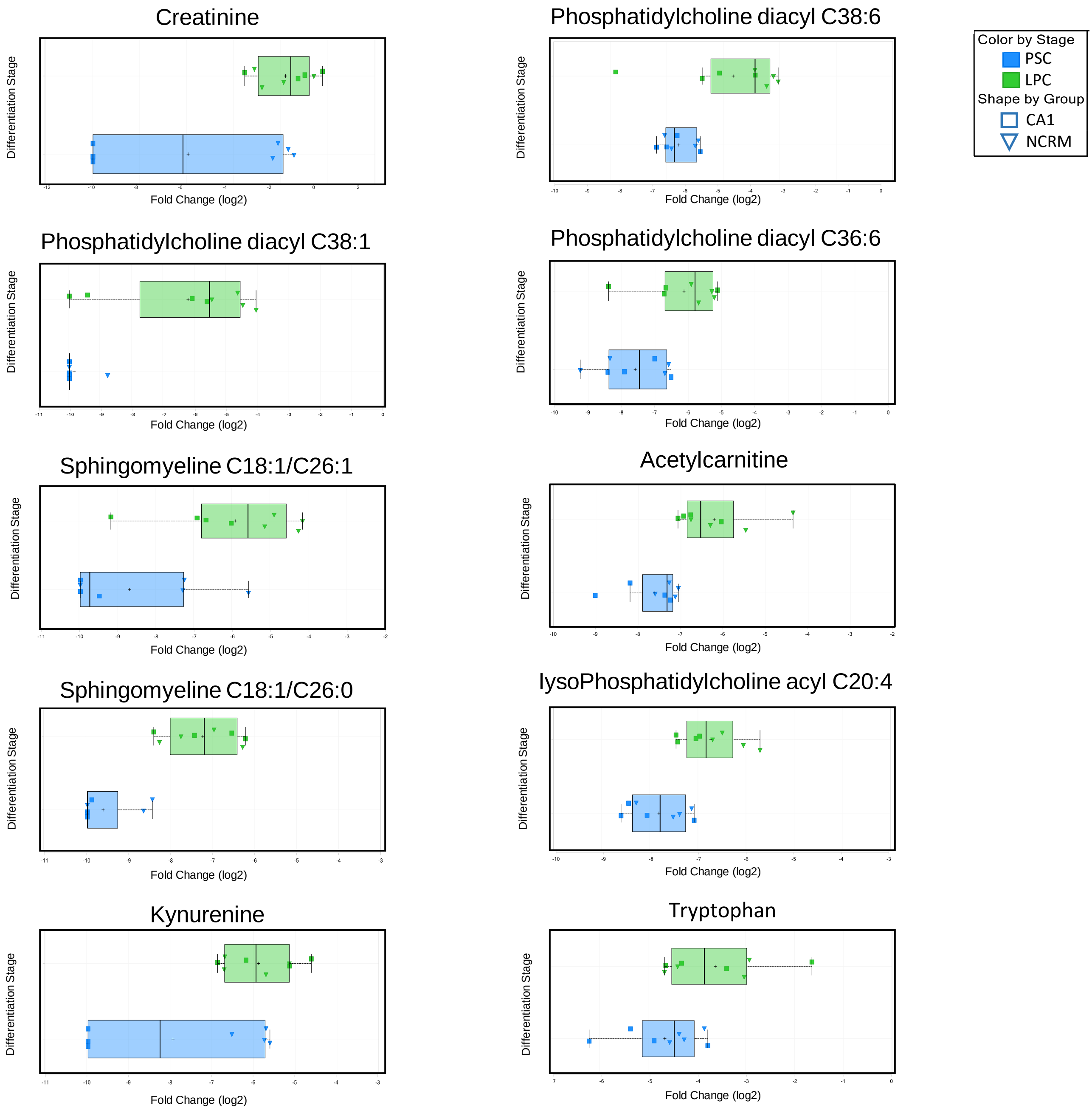

Figure S4: Box and whisker plots of significantly up-regulated metabolites from PSC to LPC of wild-type cell lines. Distributions are shown as boxplots where the central line is the median concentration, the edges of the box are the 25th and 75th percentiles and outliers are defined as 1.5 times the interquartile range and highlighted by +. Y-axis is divided into wt vs hiPro133 (green bars=LPC and blue bars=PSC). Y-axis is the log2-adjusted expression grouped per differentiation stage and cell line. *P < 0.05; data are represented as Mean ± SD for four independent differentiations, n = 4)

S5 WT Down-regulated metabolites list:

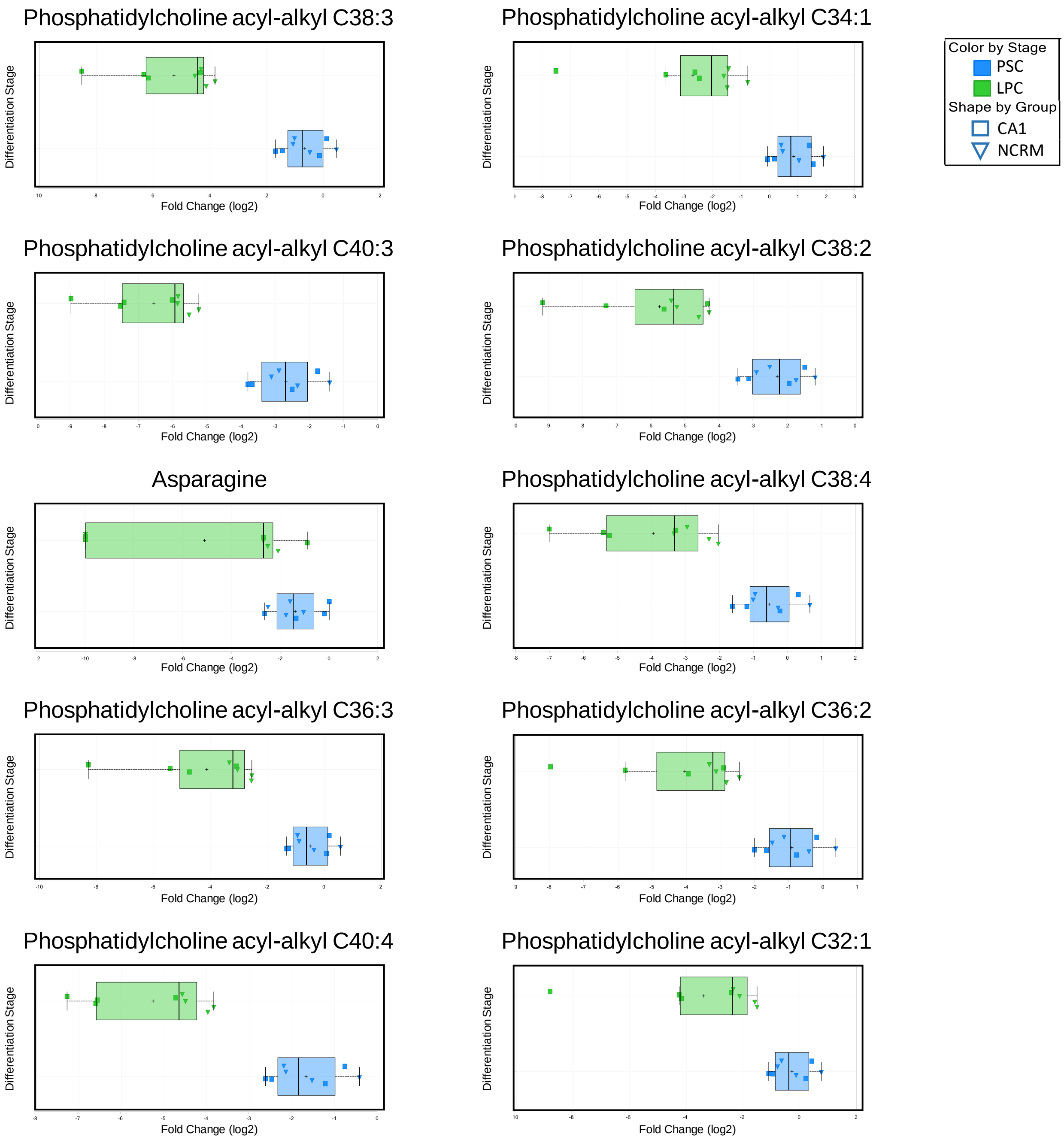

Figure S5: Box and whisker plots of significantly down-regulated metabolites from PSC to LPC of wild-type cell lines. Distributions are shown as boxplots where the central line is the median concentration, the edges of the box are the 25th and 75th percentiles and outliers are defined as 1.5 times the interquartile range and highlighted by +. Y-axis is divided into wt vs hiPro133 (green bars=LPC and blue bars=PSC). Y-axis is the log2-adjusted expression grouped per differentiation stage and cell line. *P < 0.05; data are represented as Mean ± SD for four independent differentiations, n = 4)

S6 hiPro133 Up-regulated metabolites

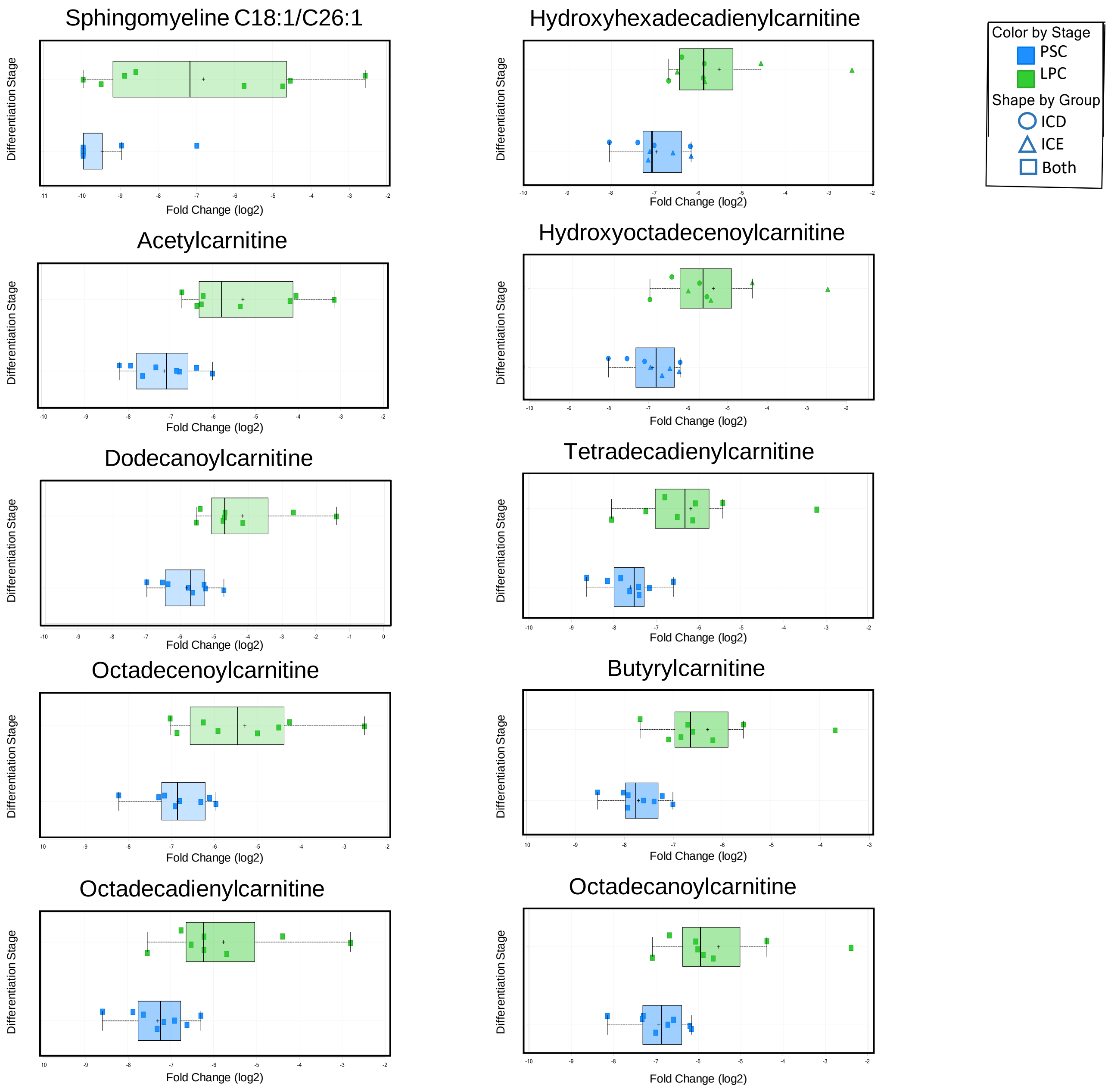

Figure S6: Box and whisker plots of significantly up-regulated metabolites from PSC to LPC of hiPro133 cell lines. Distributions are shown as boxplots where the central line is the median concentration, the edges of the box are the 25th and 75th percentiles and outliers are defined as 1.5 times the interquartile range and highlighted by +. Y-axis is divided into wt vs hiPro133 (green bars=LPC and blue bars=PSC). Y-axis is the log2-adjusted expression grouped per differentiation stage and cell line. *P < 0.05; data are represented as Mean ± SD for four independent differentiations, n = 4)

S7 KO Down-regulated metabolites

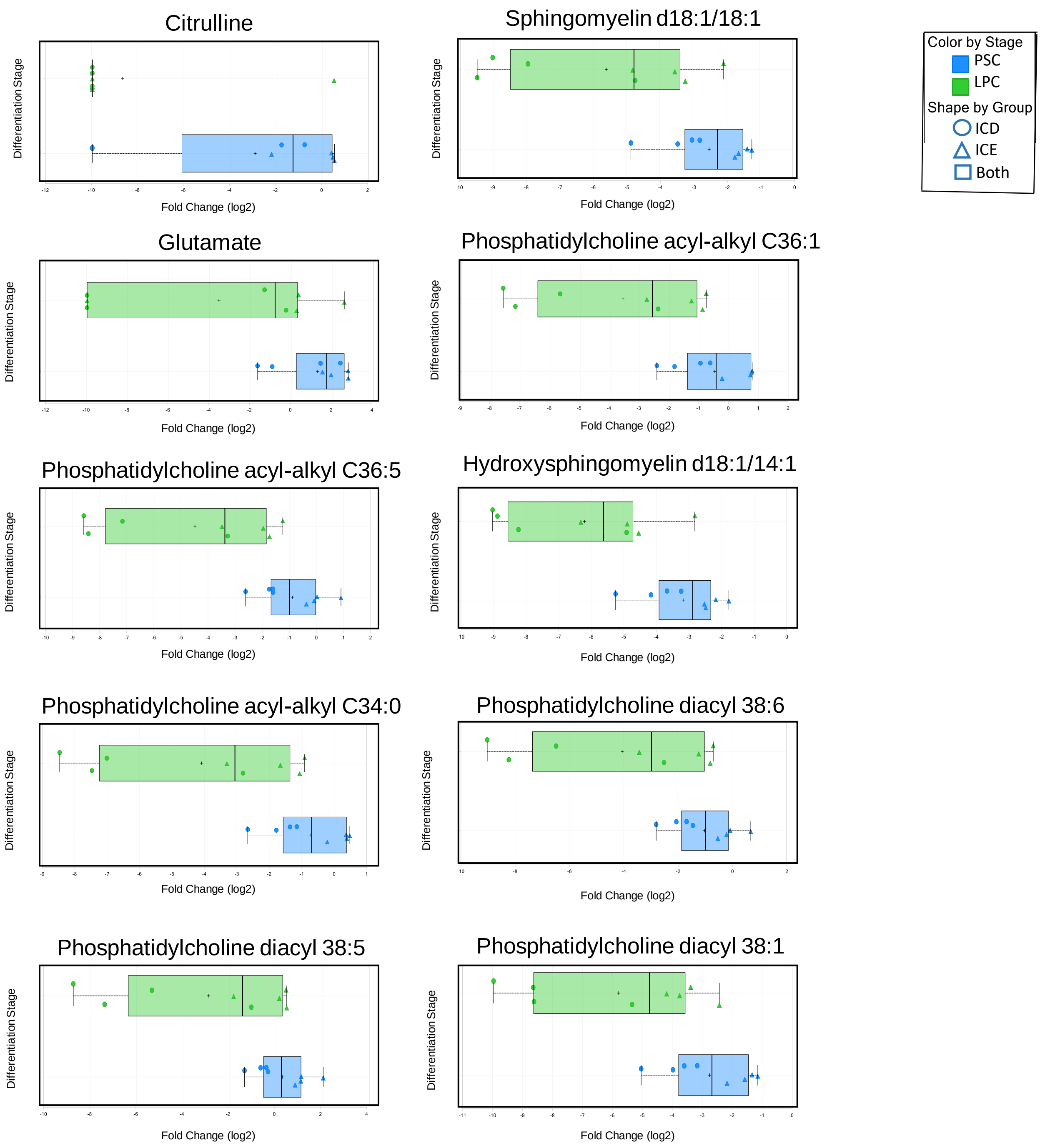

Figure S7: Box and whisker plots of significantly down-regulated metabolites from PSC to LPC of hiPro133 cell lines. Distributions are shown as boxplots where the central line is the median concentration, the edges of the box are the 25th and 75th percentiles and outliers are defined as 1.5 times the interquartile range and highlighted by +. Y-axis is divided into wt vs hiPro133 (green bars=LPC and blue bars=PSC). Y-axis is the log2-adjusted expression grouped per differentiation stage and cell line. *P < 0.05; data are represented as Mean ± SD for four independent differentiations, n = 4)

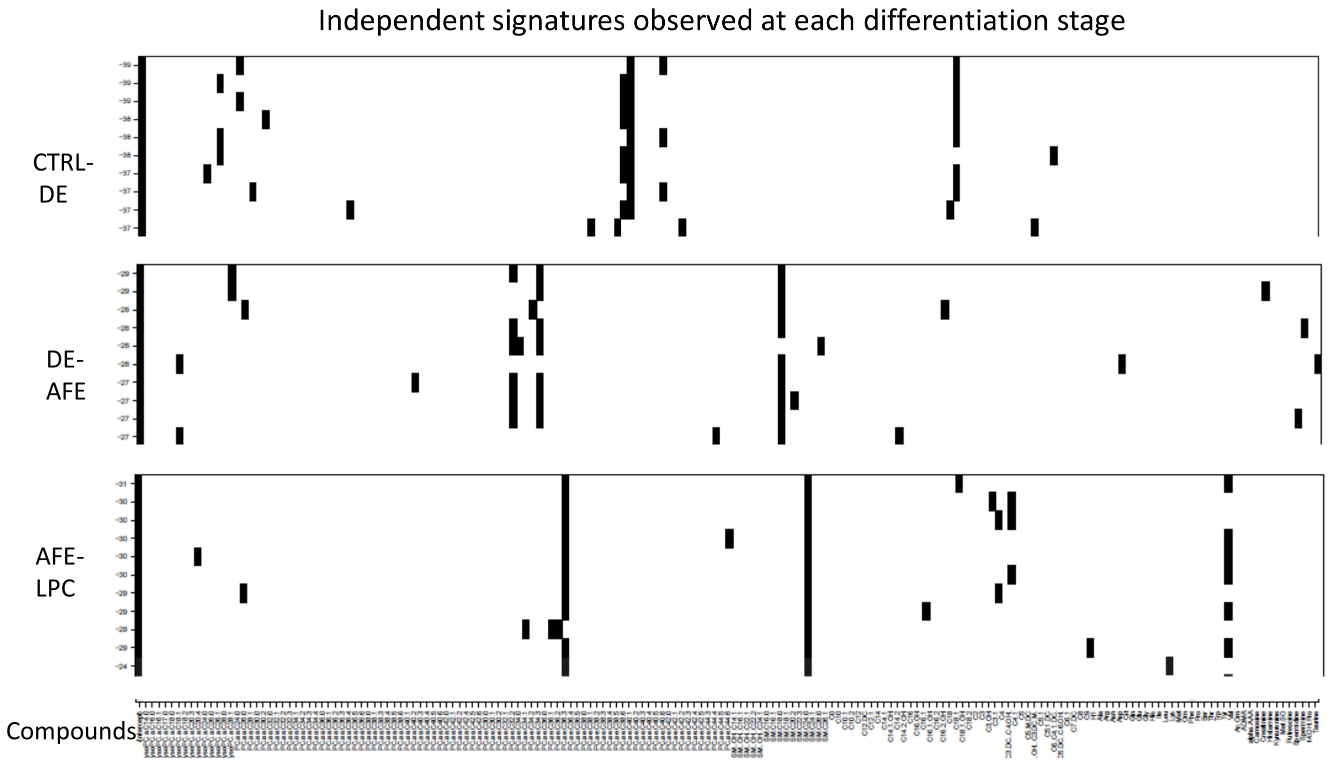

Figure S8: BIC plots with top signatures metabolites based on differentiation stage from wild-type and hiPro133 cell lines combined. BIC plots divided up into differentiation stages PSC to DE, DE to AFE and AFE to LPC. SC=Stem Cell; DE=Definitive endoderm; AFE=Anterior foregut endoderm; LPC=lung progenitor cell.
